# The Discriminatory Potential of Modern Recruitment Trends—A Mixed-Method Study From Germany

**DOI:** 10.3389/fpsyg.2021.634376

**Published:** 2021-10-25

**Authors:** Esther Kroll, Susanne Veit, Matthias Ziegler

**Affiliations:** ^1^WZB Berlin Social Science Center, Berlin, Germany; ^2^Department of Psychology, Psychological Diagnostics, Humboldt-Universität zu Berlin, Berlin, Germany; ^3^Deutsches Zentrum für Integrations- und Migrationsforschung (DeZIM-Institut), Berlin, Germany

**Keywords:** discrimination, recruitment, marginalized groups, social network sites (SNSs), active sourcing, recruitment assignment, MODE-model, far-right attitudes

## Abstract

People from marginalized groups are often discriminated against in traditional recruitment processes. Yet as companies faced with skill shortages change their recruitment strategies, the question arises as to whether modern recruitment trends such as the use of professional social network sites, active sourcing, and recruitment assignment to external agencies are affected by implicit or explicit discrimination. In our mixed-method study, we first conducted expert interviews with different types of recruiters to explore the potential for discrimination in the modern recruitment process. We then analyzed panel data from the Institute for Employment Research (IAB) in Germany to see whether there is quantitative evidence of discrimination in modern recruitment. A content analysis of the interviews shows that active sourcing and assignment of recruitment to private agencies are potentially affected by explicit discrimination. We identified three sources of discrimination in personnel selection: recruiters’ own attitudes, explicit instructions from managers, and the recruiters’ assumptions regarding companies’ preferred candidates. The results of mixed multilevel analyses with the company as a second level resonate with the qualitative findings: companies actively approach female employees, older employees, and employees who are born in Southern/Eastern Europe less often and offer women jobs less often. The effects for gender were still significant when we included far-right voting as a moderator variable on the employee level, but the interactions were not significant. Effects for gender and older people in active sourcing were also significant and robust when controlling for income, number of children, level of school completion, and educational background. Our findings suggest that current legislation may be insufficient to protect candidates who belong to marginalized groups from discrimination in modern recruitment.

## Introduction: Discrimination in a Changing Recruitment Environment

From various correspondence tests, we know that applicants who belong to marginalized groups have a higher likelihood of being discriminated against (Lane, 2016; Zschirnt and Ruedin, 2016; Quillian et al., 2017; Baert, 2018). While researchers found clear evidence for hiring discrimination, the question of what cognitive mechanism drives this discrimination in the classic application process is still an open one. In addition, the recruitment environment is currently changing. A substantial number of companies are using more active strategies and Internet recruitment to fill job openings (Roulin and Levashina, 2019). This raises the question of whether new forms of recruiting strategies are affected by discrimination as well. We conducted a mixed-method study to focus on both issues: (a) the cognitive underpinnings of hiring discrimination, and (b) discrimination in new forms of recruitment strategies. The corresponding research question reads as:

*RQ: To what extent are modern recruitment processes affected by discrimination against candidates who belong to marginalized social groups?*

Discrimination is defined as “harmful actions toward others because of their membership in a particular group” (Fishbein, 2002, p. 6). Discrimination violates the fundamental principle of equal treatment, which is protected by the Basic Law of the Federal Republic of Germany—the equivalent to a national constitution (Bundestag, 1949). In this law, it reads in Article 3(3) that “No person shall be favored or disfavoured because of sex, parentage, race, language, homeland and origin, faith, or religious or political opinions. […]” (Bundestag, 1949). Additionally, the German Bundestag, Germany’s federal parliament, passed the General Equal Treatment Act (GETA) in 2006 (Bundestag, 2006). This law protects the rights of people who are affected by discrimination. In hiring decisions, employers have now the burden of proof (Bundestag, 2006). When someone feels they have been discriminated against in a hiring decision the company must therefore be able to prove that their recruitment process has been fair. German law also assigns certain rights to a company’s works council. Every final selection must be approved by the works council (Bundestag, 1972). If a works council suspects that a shortlist is discriminatory, they can demand that more diverse job candidates be included.

With regard to the first issue, the cognitive underpinnings of hiring discrimination, previous research has shown that hiring discrimination is partially based on implicit prejudices that affect recruitment decisions automatically. To study this, researchers have often used indirect measures such as the Implicit Association Test (Greenwald et al., 1998). These studies have shown that recruiters’ selections were partially affected by implicit prejudices against ethnic groups (Agerström and Rooth, 2009; Derous et al., 2009; Rooth, 2010).

Our study contributes to this line of research by examining whether or not selection based on modern recruitment strategies is implicitly driven/automatic or explicitly driven/controlled.

Implicit associations and explicit propositions are two basic assumptions that can drive discriminatory behavior: implicitly driven or automatic discrimination, and explicitly driven or controlled discrimination. According to Bargh (1994), an automatic behavior starts involuntarily, cannot be stopped voluntarily, is unconscious, and requires low cognitive and time resources. It is not, however, necessarily unconscious (Gawronski et al., 2006). In line with this reasoning, Fazio (1990) developed the MODE model (“motivation and opportunity as determinants of the attitude-behavior relation”; see also Olson and Fazio, 2008), which proposes that people are only able to behave in a controlled or explicitly driven manner if they are motivated and have sufficient opportunities to control their automatic behavior. Time pressure and cognitive overload reduce the opportunity to engage in controlled behaviors such as deliberative decisions, and increase the likelihood of implicitly driven behavior.

Previous research often used the elaborated likelihood model (ELM) (Petty and Cacioppo, 1986) to study implicit influences in hiring, for instance, from an applicant’s perspective (Dineen and Soltis, 2011). Both the ELM and the MODE model belong to the general class of models that describe automatic and controlled behavior. The ELM model suggests that information that is less deeply elaborated has a higher likelihood of affecting behavior automatically. Nevertheless, the MODE model has two key advantages that make it a valuable alternative theoretical approach. A first advantage is that the MODE model avoids the difficulty in determining whether information is deeply elaborated because it uses time and cognitive constraints as pre-conditions for implicitly driven behavior. In interviews, we are able to ask recruiters about the amount of time they spend on each task and about general structural features of the modern recruitment process. This provides an indirect way to estimate the extent to which potentially discriminatory selections were made implicitly. A second advantage of the MODE model is that it takes into account past research on automatic and controlled behavior that has challenged the assumption that there are two distinct attitudes—implicit and explicit. Recent research suggests instead that there is just one attitude, but also a cognitive or motivational mechanism that might control automatically driven behavior. The MODE model incorporates this idea.

The consequences of explicitly and implicitly driven recruitment behavior will differ. When recruitment decisions are implicitly driven, recruiters will have less voluntary control over their selections. Yet it might be possible to prevent discrimination by modifying the recruitment procedure: for instance, by reducing time pressure or defining and implementing more structural decision-making rules. When recruitment decisions are explicitly driven, recruiters are able to control for potentially discriminatory decisions in the selection of job candidates. In this context, diversity management approaches may help to foster anti-discriminatory motivations in recruiters.

With regard to the second issue, discrimination in new forms of recruitment strategies, recruiters currently use strategies that are not covered by classic correspondence tests. Correspondence studies are the gold standard for detecting discrimination (OECD, 2013) but they can only be used with classic job application processes. Our study, however, examines whether there is evidence of discrimination in modern recruitment approaches. We focus on the discriminatory potential of three modern recruitment strategies: screening profiles on social network sites (SNSs), active sourcing, and recruitment assignment to outside recruitment agencies.

Although an abundance of training programs for active sourcing and e-recruitment can be found on the Internet, research on modern recruitment approaches such as active sourcing and the use of SNSs is still very limited (Coverdill and Finlay, 2017; Roulin and Levashina, 2019). Nevertheless, there is high interest in this subject. In addition, researchers have frequently called for a focus on questions of fairness and systematic exclusion when studying modern recruiting approaches (Anderson et al., 2004; Fountain, 2019).

In the classic recruitment process, recruiters most often use CV information to screen candidates (Robertson and Smith, 2001; Cole et al., 2007). In modern recruitment, recruiters can also screen candidates using SNSs. The common feature of SNS is that they contain web-based profiles of people whose aim is to connect with others (boyd and Ellison, 2007). Professional SNS have a different function in the recruitment process than private SNS. While decision makers use private SNS like Facebook to search for information on candidates (Ziegler et al., 2012), professional SNS like XING and LinkedIn are important in earlier recruitment stages (Roulin and Levashina, 2019). They provide as much information as CVs and are often structured similarly to CVs. Roulin and Levashina (2019) showed that profiles on professional SNS show high validity when comparing the personality assessments of profile owners and recruiters. However, screening in general is shown to be prone to automatic decisions (Bäckström and Björklund, 2017). Therefore, selections might be affected by implicit discrimination when screening on SNS is conducted under time pressure or in an unstructured way.

Social network sites are useful not just for screening but also for *active sourcing*. Active sourcing is often used when it proves difficult to find suitable job candidates. In this approach, recruiters themselves take the initiative to find job candidates with the right skills (Caers and Castelyns, 2011; Kane et al., 2014; Melanthiou et al., 2015). Active sourcing is a completely different approach than classic recruitment. Because it requires additional skills and resources, some companies prefer to assign their recruitment process to experienced agencies. This *recruitment assignment* thus constitutes a further recruitment trend that is closely related to active sourcing (Cappelli and Keller, 2014; Coverdill and Finlay, 2017). Generally, active sourcing is not new approach. It has been used for some time in headhunting for executive positions (Hamori, 2010, 2014; Cappelli and Hamori, 2014). What is new in recent years is that recruiters use active sourcing for a wide range of jobs for which they have difficulty finding candidates. Headhunting may show evidence of systematic discrimination against marginalized social group members (Dreher et al., 2011). Little is known, however, about the potential mechanisms of discrimination in active sourcing. Additionally, we know very little about the circumstances under which companies assign the recruitment process to external agencies. The question of whether the use of active sourcing or recruitment assignment is affected by implicit or explicit discrimination is still an open one.

We chose a mixed-method approach because it combines the advantages of qualitative interviews and quantitative analyses (Kelle, 2013). In a first step, we conducted expert interviews about modern recruitment processes with different types of recruiters. We aimed to explore the discriminatory potential of modern recruitment trends. Qualitative interviews are explorative in nature and not restricted to a specific set of questions. This is an advantage because it allows hypotheses to be adjusted. However, qualitative interviews are limited in generalization. Therefore, we wanted to combine the use of qualitative interviews with a quantitative study. In a second step, we analyzed panel data from the Institute for Employment Research (IAB) in Germany. Here, we sought to examine whether there is quantitative evidence of discrimination in modern recruitment processes, especially in active sourcing. These quantitative analyses allow us to draw conclusions about the generality of our propositions. However, the panel study is restricted to predefined questions and is also limited in its insights about the reasoning that underlies behavior. Taking advantage of our mixed-method approach, we combine both sources and use the insights gained from the qualitative interview study to interpret the quantitative results.

## Qualitative Interview Study

We explored the use of three recruitment approaches in qualitative interviews and assessed to what extent they are susceptible to discriminatory recruitment decisions. If we found evidence of discrimination, information on the recruiters’ resources and motivation provided further information on whether the discrimination was based on implicit or explicit cognitive processes.

One recruitment trend is profile screening on SNS. Screening in general tends to be implicitly driven or automatic rather than explicitly driven or controlled. It is important to bear in mind that recruiters’ explicit motives might differ from their implicit stereotypes or prejudices. We therefore did not use verbal expressions directly but searched for pre-conditions of implicit behavior following the MODE model. Pre-conditions for implicit behavior are a lack of resources available to decision makers to control their behavior and/or a lack of motivation to control the behavior. We were especially interested in the time available for screening and the structure of the screening process. According to the MODE model, behavior is automatic when time and cognitive resources are lacking. We assume that profile screening on SNS is conducted under time pressure and in an unstructured way.

*P1: Profile screening on SNS is conducted under limited cognitive resources and is potentially affected by automatic discrimination.*

The research on headhunting has found that recruiters approach marginalized group members less often. We are not aware of a study that focuses on the cognitive underpinnings of this unequal treatment. Coverdill and Finlay (2017) discussed in detail the importance headhunters attribute to a candidate’s fit to an organization. It appears that such decisions are driven more by the recruiter’s motivation than by time or cognitive constraints. According to the MODE model, decision makers are more likely to control their decisions when they have sufficient resources available and also when they are highly motivated to avoid mistakes. We assume that recruiters are highly motivated when they engage in active sourcing, as is the case in headhunting.

*P2: Active sourcing is conducted under high motivation and is potentially affected by explicit discrimination.*

In a similar vein, the trend toward recruitment assignment appears to increase recruiters’ motivation to select the right candidate. This trend seems to be closely related to active sourcing. We treated this as an individual trend because: (a) in-house recruiters might also use active sourcing, and (b) another motivational aspect might come into play when outside recruiters are seeking personnel. In the case of recruitment assignment, similarly to active sourcing, we assume that there are no time or cognitive constraints but a high motivation to avoid mistakes. According to the MODE model, this elicits a specific form of recruitment behavior.

*P3: Recruitment assignment to private agencies is conducted under high motivation and is potentially affected by explicit discrimination.*

### Methods and Materials

This interview study was carried out in accordance with the recommendations and ethical research guidelines of the Ethics Committee at the WZB Berlin Social Science Center. The protocol was approved by the WZB Ethics Committee in March 2017. All subjects gave their written informed consent to participate in this study.

We used snowball sampling to contact the interviewees and searched for interviewees at a job event hosted by a professional SNS in March 2017. We conducted interviews with eight experts between April and July 2017. The same interviewer was used for all interviews. Table 1 provides some descriptive information about the interviews and the interviewees.

**TABLE 1:** Sample description for the qualitative interview study.

No.	Date	In-house vs. external recruitment	Interviewee gender	Recruitment area	Description
1	April 03, 2017	In-house	Female	Recruiting in engineering	Change from external to in-house 1 year ago
2	April 20, 2017	External	Male	Executive recruiting in automotive sector	Agency founder
3	April 20, 2017	External	Male	Executive recruiting in automotive sector	Agency co-founder
4	May 10, 2017	In-house	Female	Recruiting in the automotive sector	Exclusive business partner for one automotive company
5	May 12, 2017	External	Male	Part-time agency and recruiting in the IT sector	Additional administrative support, Participatory observation
6	May 17, 2017	External	Male	Recruiting in engineering	360° recruitment
7	May 29, 2017	External	Male	Part-time agency and recruiting in the IT sector, medical and business	International recruitment and part time agency
8	July 19, 2017	External	Male	Executive recruiting IT	Agency founder

We conducted a content analysis (Mayring, 2008; Mayring and Fenzl, 2014) to analyze the interviews. We coded the interviews, first driven by theory and then by data. The five major themes of the coding were: (1) current changes in recruitment, (2) structural features of the recruitment process, (3) the process of external recruitment and in-house recruitment, (4) the use of SNS in the recruitment process, and (5) discriminatory recruitment behavior.

In the online Supplementary Material, we describe how we conducted the content analysis in detail.

### Results of the Content Analysis

#### The Structural Features of Modern Recruitment Processes

**FIGURE 1:**
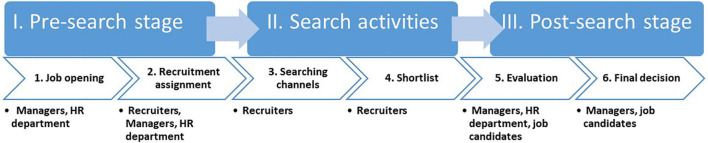
A model of the modern recruitment process. Three stages are represented in the upper boxes in white typeface. Six recruitment steps are represented in the arrows (second line) in black typeface. The main actors are listed with dots under each step.

Our analysis revealed that modern recruitment processes have a typical structure consisting of three stages (I–III) and six steps (1–6): The *pre-search stage (I)* starts with the *job opening (1)* and is followed by the *recruitment assignment (2)*. The second stage, *search activities (II)*, includes *searching channels (3)* and the *shortlisting of candidates (4)*. In the *post-search phase (III)*, the companies conduct an *evaluation (5)* of the shortlisted final candidates and make a *final decision (6)*. Figure1 shows a process model of modern recruitment processes.

#### P1: Profile Screening on Social Network Site Is Conducted Under Limited Cognitive Resources and Is Potentially Affected by Automatic Discrimination

It turned out that SNS are indeed relevant in modern recruitment processes. All interviewed recruiters use SNS on a daily basis. We therefore explored whether recruiters screen profiles on SNS under limited resources. This may increase implicit discrimination.

Our analyses suggest that the screening of profiles on SNS is a highly automatic action that does not require substantial cognitive resources. All interviewees had difficulties describing clear rules and structural procedures for how they screened a list of candidates. Recruiter i4 explained that s/he screens profiles in the same way one would carry out a Google search. In this case, s/he admitted that s/he might sort out Indian candidates faster.

This gives evidence that profile screening on SNS might be prone to implicit discrimination. But this evidence is limited to only a few recruiters. Generally, we found very little evidence in support of the assumption that profile screening on SNS is affected by implicit discrimination, for instance, by ethnicity, age, or gender.

Hence, profile screening might not be the most important purpose for which recruiters use professional SNS. Many recruiters explained that they seldom screen profiles on professional SNS. They admitted that their search on SNS is often limited to a handful of candidates and sometimes is even unsuccessful. According to recruiter i6, many employers are searching for employees with specific qualities, and the pool of qualified candidates is very small:

*(1) And I have had requests where I just entered the name of the position and Berlin and Brandenburg and got zero hits—that happens too. (i6)*

When recruiters look at the profiles on professional SNS platforms, they seem to have no time pressure. The recruiters we interviewed often described taking several minutes to get a first impression of each candidate. Recruiter i7 explained that the amount of time spent reading a profile depends on how much information a candidate provides. When reading time depends on the amount of information, it seems that there is no time pressure. This is evidence that recruiters might have sufficient opportunities to control their implicit preferences.

On this basis, we must reject Proposition 1. Although profile screening on SNS might be affected by discrimination, it appears not to be important for modern recruitment processes.

#### P2: Active Sourcing Is Conducted Under High Motivation and Is Potentially Affected by Explicit Discrimination

Interviewees described a shift from traditional to active sourcing approaches. For many job openings, passive sourcing activities seem to be insufficient. Job advertisements attract too few applicants, even when they are published on professional platforms. Many recruiters therefore prefer to actively contact suitable candidates, whether those candidates are currently searching for a new job or not. When recruiters want to make contact with suitable candidates, they use an active sourcing channel.

Recruiters often use their own professional networks as a first step when they get a new recruitment assignment, especially when recruiting executive personnel. Our analysis revealed that the use of these exclusive networks may discriminate against some candidates. It seems that not everyone has access to these kinds of networks. Recruiter i8 explained that, officially, his/her recruitment agency is willing to work with all candidates, but—unofficially—only if they are highly qualified, if they work in the field that the agency is specialized in, and if they have several years of experience. However, recruiters often do not think that recruitment through exclusive networks prevents people outside their networks from getting fair access to jobs. Recruiter i2 felt that this was the obvious approach based on the excellent referrals his network guarantees:

*(2) The network plays a role in that sense that I have access to the network and can see: Who is in this network? Because as the old saying goes, good people only know good people. (i2)*

Only one recruiter, i3, openly discussed the exclusiveness of these agencies:

*(3) There are still old men running around who have their little black books and their networks and know how to use them. Personnel consulting in the area of executive search is a “good old company”; an old, slightly antiquated and outdated industry that lives off of personal referrals and—yes—also very particular network activities. (i3)*

Another feature is the use of keywords, which limit the pool of potential candidates. When recruiters search actively on professional platforms, they filter profiles using keywords. The keywords depend heavily on the skills required for the job. Yet some keywords systematically exclude candidates with specific personal characteristics. Recruiters commonly limit their searches to a specific geographical area. This may discriminate against candidates who do not live in urban areas or candidates who are based in a foreign country. Recruiters explained that they use geographic restrictions because they think many candidates are not flexible enough. They often sort out candidates who are not located in the area rather than asking them about their willingness to relocate:

*(4) If you know that your customer is located in a specific city, you have good reasons to give it some thought: Do I really want to ask this big question about relocating or don’t I?—Whether that’s a good idea is another question, but it would be an option. (i3)*

Another reason might be that placing no geographic restrictions on the search would lead to a pool of candidates that is too large to handle. Recruiter i6 said that no geographic restrictions would increase the search results dramatically:

*(5) If I searched on a national level using just the search term “electrical engineer,” I would get search results in the four-digit range. (i6)*

The analysis suggests that recruiters are fully aware that this restriction can potentially exclude some candidates who are equally qualified.

#### P3: Recruitment Assignment to Private Agencies Is Conducted Under High Motivation and Is Potentially Affected by Explicit Discrimination

Interviewees reported that active sourcing is time-consuming and expensive. But in their opinion, it sometimes is the only way to fill a job opening. The business model of external recruitment agencies generally involves being specialized in sectors with a high demand for labor, such as engineering and IT. Some recruiters believed that a recruitment agency is able to recruit qualified personnel better than internal human resources (HR) departments.

Recruiters from external agencies are able to use some active sourcing strategies that in-house recruiters do not use, for instance, headhunting. Several of the recruiters interviewed thought that the possibility of headhunting is an important reason for companies to have external agencies do the recruiting. Headhunting has a negative image and is often considered unethical. Companies therefore often hire external agencies for this task. In-house recruiter i4 stated that his/her company sometimes outsources headhunting to external recruitment agencies:

*(6) Headhunting means poaching people directly, calling them in the office at a competing company. We just can’t do it because it can damage our image. (i4)*

Our analysis suggests that recruitment by external agencies is potentially affected by explicit discrimination. One reason is that external recruiters often get paid only if recruitment is successful. External recruitment agencies carry the entire financial risk. This implies a high level of economic pressure on the individual external recruiter as recruiter i6 explained:

*(7) We work, for instance, like almost—or like the great majority of personnel consultants, based on our success. That means we are only rewarded if we fill an open position. The company does not bear any financial burden when they bring personnel consultants on board. (i6)*

Another reason is that external recruiters are especially motivated to avoid mistakes in their shortlists. They try to avoid a feedback loop in the recruitment process because this would be a waste of time and money. Recruiters are highly motivated to understand what the company “really” wants:

*(8) We wouldn’t be able to carry out our task correctly or we wouldn’t be able to fulfill it if we were presenting candidates and didn’t know that they would end up running into a glass ceiling. (i8)*

External recruiters feel bound to fulfill companies’ expectations. They explained that the economic pressures leave them no space to select candidates who violate these expectations. As a consequence, recruiters only select candidates who closely resemble the stereotypical “ideal” candidate. This selection might be biased by availability heuristics (What types of people work for this company? What candidates did this company hire recently?) as well as representativeness heuristics (What is the probability that the company will approve my shortlist?) (Tversky and Kahneman, 1974). These expectations and the preferences of managers therefore involve a high risk of making discriminatory selections.

#### Three Sources of Discrimination

In analyzing the interview data, we identified three sources of discrimination: recruiters’ own attitudes, managers’ explicit instructions to exclude certain candidates, and recruiters’ assumptions about managers’ preferences.

##### Recruiters’ Attitudes

The first source of discrimination is the recruiters’ own attitudes, which may potentially motivate recruiters to exclude certain candidates. Recruiters often seem to evaluate candidates based on their first impressions. This is a form of stereotypical decision. Candidates’ photographs, for example, are very important for recruiters’ first impressions. All recruiters emphasized that a “business look” is the most important aspect of professional photos. When recruiters see private photos, they doubt the candidate’s professionalism.

Recruiters often evaluate whether or not a candidate’s profile provides “complete” information: gaps raise suspicion. When candidates cannot reasonably explain the gaps in their CVs, recruiters interpret this as an indication that the candidate lacks professionalism. Recruiter i5 explained that s/he strictly sorts out candidates with gaps in their CV:

*(9) I have to admit, maybe sometimes I don’t give those people the chance they deserve. But if someone has a sizeable gap in there [in his/her CV] and there is no further education, nothing, then I feel like—[…] the commitment is lacking, and they’re out! Or things like a 3-year sabbatical! That, to me, is actually like a red flag to a bull. (i5)*

However, we found very little evidence that recruiters generally have negative attitudes toward job candidates from certain social groups, such as ethnic minorities, women, or older candidates. Only one of the recruiters revealed negative attitudes toward Indian candidates:

*(10) It’s often the case among Indian applicants—and I’ve been told this by my department as well, also by the IT department, who work a lot with consultants in India—that loyalty to the company is extremely different there. In India, if someone earns a few euros more at a different company, he will change employers. (i4)*

##### Managers’ Explicit Instructions to Exclude Certain Job Candidates

The second source of discrimination is the explicit instruction by managers to exclude candidates from certain social groups. Almost every interviewee reported that at some point in time, a manager or HR department staff member stated explicit preferences for candidates’ gender, age, ethnicity, or country of origin—for instance, recruiter i7:

*(11) And we have some companies—or we have HR or other departments— clearly saying: I only want a woman. Or: I only want a man. Or sometimes they also state a specific age. (i7)*

According to our interviewees, managers express gender preferences in both directions: Some want female employees, especially in very technical and male-dominated areas. Others want male candidates. Many interviewees considered gender preferences to be a valid and not discriminatory recruitment criterion. Recruiter i5 stated that in male-dominated areas, companies hope to improve the working environment by hiring more women:

*(12) There are often clients who say straight out: I have too many men here. It’s so—it makes things so rude and crude. It might be nice to have a woman on the team again. For instance, so the burping would stop, or so people would clean up their desks again, or things like that. (i5)*

Recruiters explained that knowing companies’ preferences is essential for the recruitment success. Often recruiters ask explicitly about some characteristics, for instance, age, gender, and regional preferences. They ask the question about preferences as casually as possible, as recruiter i3 reported:

*(13) I ask, for instance: “Could it also be—does the candidate necessarily have to be male?”— Yes—“From what age upward is he no longer acceptable to your company?”—Yes, it sounds very blunt, but the more bluntly you ask, the better the answers you get. We are all very sensitive to anti-discrimination issues. (i3)*

At the same time, recruiters are aware that these preferences are discriminatory. If such preferences become public, it could have severe legal consequences and would be extremely damaging to the company’s image. Recruiter i3 admitted that companies will only express their preferences if they completely trust their business partner’s loyalty.

##### Assumptions About Stereotypical Preferences

The third source of discrimination is recruiters’ assumptions about managers’ preferences.

Interviewees commented that ethnic preferences are rare, compared to regional, age, and gender preferences. Many recruiters explained that language barriers are the only reason why ethnic minorities are sorted out. But all of the recruiters were able to relate at least one anecdote about ethnic preferences from their experience in recruiting. Recruiters admitted that managers often do not explicitly express their ethnic preferences because it is a very sensitive topic. Recruiters implicitly draw conclusions about their clients’ ethnic preferences or prejudices. According our analysis of recruitment processes, recruiters draw conclusions mainly from two situations: their meetings when being given the recruitment assignment and the company’s explanations for why they rejected a candidate who seemed suitable to the recruiter. Recruiter i3 put it like this:

*(14) There are companies where you cannot present a person of color as a candidate. And the company absolutely insists that there are rational reasons for that. But you don’t believe it. (i3)*

The second recruitment step seems to be critical for the recruiters’ impressions of managers’ preferences. Recruiters use these meetings not only to get to know what skills are needed for a position or what kind of budgets are available for recruitment, but also to get an idea about the preferred characteristics of the job candidate. If a company has a very conservative atmosphere, recruiters may assume that the company’s managers have reservations about employing ethnic minorities. Our analysis showed that recruiters do not always wait for managers to reveal their personal preferences explicitly. If recruiters have a strong sense of hidden recruitment agendas, they will ignore some candidates to avoid management rejecting their shortlist. Recruiter i5 admitted that s/he adjusts his/her search behavior to ethnic stereotypes on his/her own when companies are located in Eastern Germany—an area where very few immigrants live and where many people have negative attitudes toward immigrants:

*(15) If I’m searching for candidates for a company in Mecklenburg-Western Pomerania, for instance, I won’t be able to approach the task in such a multicultural way, because I’ll get more “nos” than “yeses.” That means, depending on the region, I sometimes look at where the companies are from. And then—unfortunately—I have to exclude some great candidates who actually would have fit. (i5)*

Note, however, that it remains unclear whether companies in Eastern Germany actually have ethnic prejudices or if the recruiter has a stereotypical image of companies in Eastern Germany. We consider this a meta-cognitive process.

### Discussion of the Qualitative Results

Our qualitative interviews suggest that the modern recruitment approaches “active sourcing” and “recruitment assignment” are potentially affected by explicit discrimination. Recruiters are highly motivated to exclude certain candidates. At the same time, we found little evidence that modern recruitment is potentially affected by implicit discrimination. Screening on SNS might be implicitly driven because it often is unstructured. But recruiters rarely screen candidates on SNS. They tend to use SNS for active sourcing instead. We identified three sources of discrimination that potentially motivate recruiters to systematically exclude marginalized candidates: the recruiters’ own attitudes, the managers’ instructions to exclude certain candidates, and the recruiters’ assumptions about managers’ preferences.

#### Old-Fashioned Discrimination in Modern Recruitment Trends?

Our results show that modern recruitment trends such as active sourcing and recruitment assignment to external agencies are potentially affected by discrimination against marginalized candidates. More generally, profile screening seems to be of minor importance in modern recruitment processes. However, professional SNS appear to be important for active sourcing. In active sourcing, recruiters are motivated to reduce the pool of candidates, for instance, by limiting the search geographically or by searching through personal networks. But these active sourcing practices may exclude some candidates systematically, for instance, those who live outside major urban areas or who are outside the established networks of people in power. The same holds true for recruitment assignment by external recruiters, who work under relatively high economic pressure. External recruiters are therefore motivated to avoid taking risks in their shortlists, which in turn increases the likelihood of discrimination, for example, if recruiters believe that the company has discriminatory preferences.

Many interviewees explained that discrimination does not play an important role in hiring. Yet not only do recruiters apparently take candidates’ qualities into account; all participants were also able to describe stereotypical and prejudicial preferences in recruitment processes that potentially lead to a discriminatory selection. We found little support for the idea that modern recruitment processes are potentially affected by implicit discrimination. The reason is that screening, which might be done in an unstructured way, does not play an important role in modern recruitment. However, we found high support for the idea that recruitment trends may be affected by explicit discrimination. Recruiters seem to be motivated to engage in discriminatory decisions, and they do not seem to lack the opportunities to control for automatic influences. This implies that recruiters are able to control discriminatory decisions, for instance, due to gender, ethnicity, or age. Thus, our results point more in the direction of old-fashion racism or blatant prejudices than toward modern racism (Devine, 1989) or subtle prejudices (Pettigrew and Meertens, 1995; Coenders et al., 2001).

#### Own Stereotypes, the Others’ Stereotypes and Meta-Stereotypes as Drivers

We found three sources of discrimination. The first source consists in the recruiters’ own stereotypical selections. These are often based on the recruiters’ first impressions of the candidates. Photographs and a gap-free CV are very important for these first impressions. Beside this, we found very little evidence that first impressions are based on recruiters’ personal preferences. The second source is the managers’ explicit preferences for some candidates. This seems to be a major reason why recruiters exclude minority candidates. Recruiters and managers are aware that such preferences are illegal and may have severe consequences. However, recruiters feel responsible for keeping these preferences strictly confidential. The third source of discrimination consists in recruiters’ assumptions about managers’ preferences. These impressions may motivate recruiters to explicitly exclude candidates who they think do not fit the client’s preferences. Ethnic preferences fall into this category due to the sensitivity of racial prejudices.

While previous research has tackled the first two sources of discrimination (Brief et al., 2000; Petersen and Dietz, 2005, 2000; Carlsson and Rooth, 2012; Derous et al., 2012), the last source has been studied relatively little in the context of recruitment. Brief et al. (2000) pointed out that discrimination in hiring is sometimes based on “obedience to authority.” In their laboratory setting, participants adapted their selections to accommodate their client’s assumed negative attitudes toward marginalized people. These negative attitudes were expressed explicitly in a “client’s letter” instructing that the participant should “not hire anyone that is a member of a minority group” (Brief et al., 2000). This leaves open the question of whether recruiters would draw indirect conclusions about company’s preferences as well. Future recruitment research might focus on these metacognitive processes (Greifeneder and Schwarz, 2014) in hiring.

## Quantitative Panel Study

Exploring the cognitive mechanism of the decision-making process was the first step to assess discrimination in modern recruitment. We found initial evidence that recruitment through active sourcing and recruitment assignment are potentially affected by discrimination. In a second step, we wanted to investigate whether there is quantitative evidence that active sourcing discriminates against employees from marginalized groups.

Our interview study revealed that, in contrast to the findings of Roulin and Levashina (2019), real recruiters often do not review SNS profiles systematically. Recruiters use SNS more to gain access to potential candidates—that is, for active sourcing rather than for screening. Our research looked at whether people in marginalized groups are actively sourced by recruiters less often than others. The question of whether SNS was used for this active sourcing is not important.

We therefore modified the research question. It now reads:

*RQ: To what extent is active sourcing affected by discrimination against candidates who belong to marginalized social groups?*

Research on headhunting shows that members of some social groups are headhunted less often than others. We assume that active sourcing might show similar effects because active sourcing is carried out in a very similar way to headhunting. While headhunting is used prominently in executive search, active sourcing is applied to a broader range of jobs where there are labor shortages. We focused on three sociodemographic characteristics that were often mentioned in our interviews as potential criteria for excluding candidates: age, gender, and migration background. When marginalized group members are actively sourced less often, we consider this evidence that active sourcing as a recruitment trend might be affected by discrimination.

H1: Candidates from marginalized groups are actively sourced less often than candidates who do not belong to marginalized groups.

H1a: Older candidates are actively sourced less often than younger candidates.

H1b: Female candidates are actively sourced less often than male candidates.

H1c: Candidates with a migration background are actively sourced less often than native German candidates.

As a second outcome, we studied the extent to which marginalized group members are offered jobs less often than non-marginalized groups. While active sourcing is considered a search strategy, this variable gives valuable information on potential unequal treatment in hiring decisions. This variable differs from active sourcing. Here, we compare people who were offered jobs to people who were not offered jobs. It is not important whether these people were actively sourced or whether they applied for the jobs themselves. Again, we focused on the three sociodemographic characteristics: age, gender, and migration background. Through this approach, we are able to compare whether it is only active sourcing that is affected by discrimination or whether job application processes are potentially affected as well.

H2: Candidates from marginalized groups are offered jobs less often than candidates who do not belong to marginalized groups.

H2a: Older candidates are offered jobs less often than younger candidates.

H2b: Female candidates are offered jobs less often than male candidates.

H2c: Candidates with a migration background are offered jobs less often than native German candidates.

The interview study revealed that recruiters take into account where the company is located. They use this information to draw conclusions about the companies’ openness to marginalized candidates. Therefore, we examined whether effects were moderated by the regional share of far-right voting. Previous research found a relationship between far-right support and negative attitudes toward candidates from some marginalized groups, such as people of foreign descent and women (Hogan and Haltinner, 2015; Träbert, 2017; Donovan and Redlawsk, 2018; Mills et al., 2020). Studies have also reported an association between far-right attitudes and negative attitudes toward older people (Sigelman and Sigelman, 1982; Henry et al., 2019). Van Assche et al. (2017) argued, however, that this is counter-intuitive, and that far-right attitudes are intuitively associated with an appreciation of older people. When recruiters make assumptions about managers’ preferences, they might share the same intuition. Our hypothesis therefore reads:

H3: The effects of age, gender, and migration background are moderated by the regional variance of far-right voting.

H3a: Older candidates are actively sourced and offered jobs more often in regions with a high share of far-right voting.

H3b: Female candidates are actively sourced and offered jobs less often in regions with a high share of far-right voting.

H3c: Candidates with a migration background are actively sourced and offered jobs less often in regions with a high share of far-right voting.

### Methods and Materials

Quantitative data on recruiting trends is limited. Fortunately, the Institute for Employment Research (IAB) in Germany, which hosts the data from the German Federal Employment Agency (BA), collects panel data on personnel and recruitment issues.

#### The Linked Personnel Panel Data

We used the Linked Personnel Panel (LPP) to address our research question (Mackeben et al., 2018; Schütz et al., 2018; Tschersich and Gensicke, 2018). This data allowed us to make quantitative estimates of how often marginalized social groups were approached when companies applied active sourcing.

The LPP is a panel study that enables the linkage of employer and employee data. Data were gathered in structured interviews. The panel data are available for three waves: 2012/13; 2014/15; 2016/17. Our main dependent variables (DVs) were from Wave 2 (2014/2015) and 3 (2016/2017) of the employee panel. Additionally, we added Wave 1 (2012/2013) to complete the employees’ personal data, such as age and country of birth. We also used the employer panel data and the IAB Establishment Panel for sample description and to consider far-right voting in the company’s geographic area. We restricted the data to the first answer of an employee when a person answered our DVs in Waves 2 and 3 to avoid overestimation.

Employees were interviewed by telephone. Most of the employees interviewed were part of the panel. Refresher samples and the first wave were selected based on the companies’ employment history (Schütz et al., 2018). The companies were the same as in the IAB Establishment Panel. The companies are a random but disproportional sample, stratified by company size, economic sector, and region (Mackeben et al., 2018).

#### Analytical Strategy

The LPP employee survey asked respondents in the second and third waves of the study whether those who intended to change jobs had searched for a new job or had been approached by another company. We consider this a question about active sourcing. People who answered that other companies had approached them were actively sourced. People who answered that they “actively” searched themselves took the classic approach. Everyone who answered the question about active sourcing was also asked whether or not they had been offered a job by another company. We consider this an attempt to entice an employee to another company and used this question as a second DV.

We conducted our analysis in four steps. In the first, we calculated the null models, followed by mixed logistic regressions in the second step. In the third step, we calculated interactions between our independent variables (IVs) and far-right voting. In the fourth and final step, we included four control variables to our initial mixed logistic models to see how robust the effects are. In each step, we conducted separate analyses for the two DVs.

We analyzed two DVs: whether people were approached actively by another company (DV1: active sourcing) and whether they were offered a new job (DV2: job offer). Both variables are dichotomous. We used four IVs that describe marginalized groups: the employee’s age (IV1), gender (IV2), the employee’s country of birth (IV3), and the employee’s citizenship (IV4). In the literature, the threshold for discrimination against older people is often considered to lie between 45 and 55 years (Macdonald and Levy, 2016). Therefore, we studied age effects both as a continuous variable and as a dichotomous variable (younger vs. older than 50 years). The employee’s country of birth (IV3) and the employee’s citizenship (IV4) refer to the employees’ migration background. The variable “country of birth” (IV3) has five categories: Germany (base), Southern/Eastern Europe, Asia, Northern/Western/Central Europe, and remaining countries. The variable “citizenship” (IV4) has three categories: German citizenship (base), German and foreign citizenship, and foreign citizenship only. For all our analyses, we used the company ID (L2V) as a second-level variable. Additionally, we were interested in whether regional differences in attitudes toward diversity moderate the effects of marginalized groups. The LPP does not include any data on the local share of attitudes. Therefore, we used far-right voting from the 2013 German federal elections as a proxy for anti-immigrant attitudes or attitudes against working women. We merged the LPP data with the IAB Establishment Panel, which is representative of the German labor market and is the host survey of the LPP, and linked the results of the federal elections in 2013 to the region where the employee’s company was located. This allows us to use the percentage of far-right voting in the 2013 federal elections in the region where the employee works. Here, we use the fact that recruiters usually restrict their active sourcing attempts to the region in which the company is located. Far-right voting includes three parties—the “NPD,” the “Republikaner,” and “Die Rechte.” The percentage of the population who voted for these far-right parties was 1.84% on average, with a minimum of 0.39% and a maximum of 5.55%. We used far-right voting as a moderator variable (MV) at the employee level.

We used STATA in its 14th edition for all our calculations (StataCorp, 2015). In addition, we used the ggplot2 package (Wickham, 2016) of R (R Core Team, 2018) to visualize the results. Data access was provided on-site at the Research Data Centre (FDZ) of the German Federal Employment Agency (BA) at the Institute for Employment Research (IAB) and subsequently through remote data access. This means that we did not have direct access to the data. All outputs were double-checked, automatically and by hand, before we were allowed to report them. In cross-tabulations, every cell with fewer than 20 people was deleted due to data protection regulations of the Institute for Employment Research (IAB).

#### Subjects

When we put all three waves of the LPP employee panel together, we had a sample of 13,999 employees. However, we had to deal with a substantial amount of missing data. The sample for active sourcing included 3,949 employees (28% of the full sample) from 1,413 companies as higher-level units (min. = 1 employee, max. = 28 employees, *M* = 2.8 employees). Concerning the second DV, job offers, the sample included 2,356 employees (17% of the full sample) from 1,109 companies as higher-level units (min. = 1 employee, max. = 18 employees, *M* = 2.1 employees). When we included far-right voting, our sample was further reduced by almost half of the available cases. The reason for this reduction is that we needed to merge the employee data with two further datasets, the LPP employer data and the IAB Establishment Panel, to get the regional district codes (German: *Kreiskennziffer*).

**FIGURE 2:**
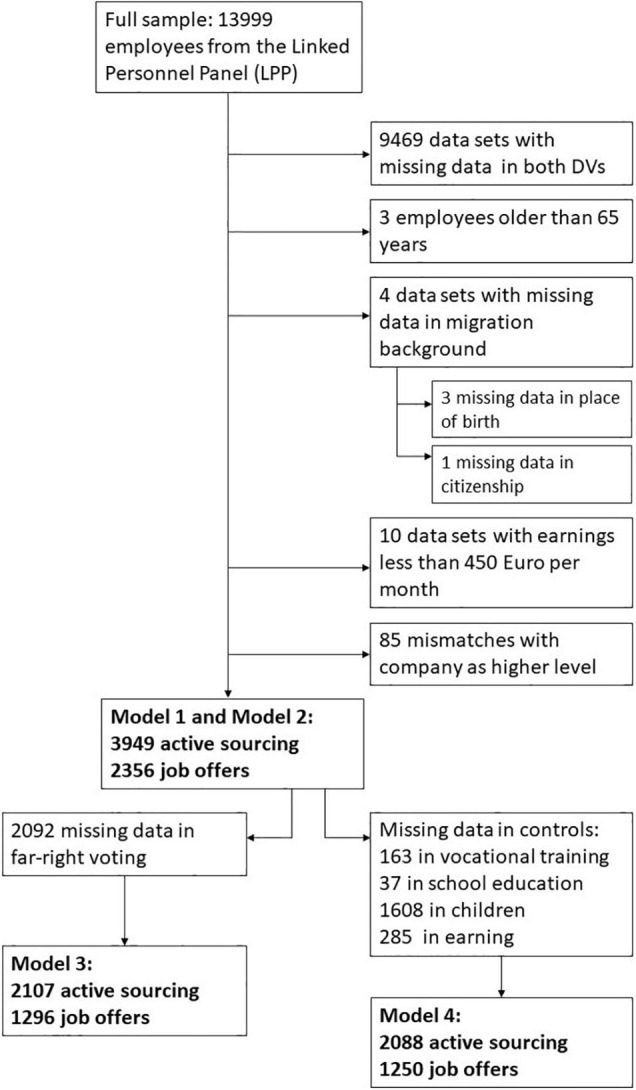
The flow chart of the quantitative sample derived from the Linked Personnel Panel (LPP). DV = dependent variable.

Figure 2 shows a flowchart on our sample.

We restricted our sample to people who were younger than 65 years because people in Germany usually retire in that age. Additionally, we restricted the sample to those who earn 450 euros or more per month. This is the typical income from mini-jobs and excludes volunteer workers. There are very few people born in Northern/Western/Central Europe and in the category “remaining countries.” For this reason, we only go into detail for Southern/Eastern Europe and Asia. Table 2 gives a sample description for the full sample and the samples of the two DVs.

**TABLE 2:** Sample description for the quantitative analyses.

Parameter	Full sample *N* = 13999	Active sourcing (DV1) *n* = 3949	Job offer (DV2) *n* = 2356
Age	*M* = 46.60, *SD* = 10.80, min. = 18, max. = 74	*M* = 43.48, *SD* = 10.32, min. = 20, max. = 65	*M* = 43.47, *SD* = 10.41, min. = 20, max. = 65
Older than 50 years	47.12%	32.31%	34.76%
**Gender**			
Male	71.18%	69.71%	74.32%
Female	28.82%	30.29%	25.68%
**Birthplace**			
Germany	90.39%	90.73%	92.06%
Southern/Eastern Europe	6.06%	5.39%	4.33%
Asia	2.26%	2.43%	1.95%
Northern/Western/Central Europe	0.69%	0.91%	–
Remaining countries	0.49%	0.56%	–
**Citizenship**			
German citizenship	94.59%	94.35%	93.97%
German and foreign citizenship	2.54%	2.56%	3.14%
Foreign citizenship	2.83%	3.09%	2.89%
Children	*M* = 0.66, *SD* = 0.88, min. = 0, max. = 6	*M* = 0.74, *SD* = 0.90, min. = 0, max. = 5	*M* = 0.79, *SD* = 0.91, min. = 0, max. = 5
Income	*M* = 2229, *SD* = 1151, min. = 1, max. = 20000	*M* = 2226, *SD* = 1163, min. = 450, max. = 20000	*M* = 2515, *SD* = 1364, min. = 450, max. = 20000
**School level completion**			
Lower secondary (Hauptschule)	23.44%	16.03%	12.95%
Intermediate secondary (Mittlere Reife)	42.99%	42.19%	39.39%
Technical secondary (Fachoberschule)	10.62%	12.74%	13.92%
Upper secondary (Abitur)	21.39%	27.83%	32.89%
**Vocational degree**			
Apprenticeship	47.97%	42.87%	34.30%
Vocational school	9.53%	8.89%	8.66%
Technical college	19.87%	20.06%	22.96%
Advanced technical college	8.81%	10.58%	12.56%
University	10.09%	14.03%	18.00%
Number of employees	*M* = 1118, *SD* = 5313, min. = 7, max. = 60261	*M* = 699, *SD* = 2745, min. = 7, max. = 60261	*M* = 801, *SD* = 3390, min. = 24, max. = 60261
Far-right voting	*M* = 1.84, *SD* = 1.07, min. = 0.39, max. = 5.55	*M* = 1.85, *SD* = 1.08, min. = 0.39, max. = 5.55	*M* = 1.84, *SD* = 1.06, min. = 0.39, max. = 5.55

*The first column refers to the full sample, the second column to active sourcing (DV1), and the third column to job offers (DV2). Empty cells account for data protections rules.*

## Results

### Main Effects for Marginalized Groups

We conducted our analyses in four steps. The full STATA output of the calculations is provided in the Supplementary Material.

Table 3 gives an overview of the calculated effects for active sourcing. Table 4 gives an overview for job offers.

**TABLE 3:** Mixed multilevel analyses for active sourcing with fixed effects for employees and random intercepts for company as second level.

Parameter	Model 1 (*n* = 3949)	Model 2 (*n* = 3949)	Model 3 (*n* = 2107)	Model 4 (*n* = 2088)
	
	Fixed effects (odds ratios)			
Intercept	0.31 (0.01)***	0.39 (0.02)***	0.34 (0.03)***	0.24 (04)***
**Level 1 (employee)**				
Older age		0.78 (0.07)**	0.84 (0.10)	0.64 (0.09)**
Gender		0.55 (0.05)***	0.61 (0.08)***	0.66 (0.11)*
**Birthplace**				
Southern/Eastern Europe		0.62 (0.13)*	0.56 (0.22)	0.62 (0.19)
Asia		0.85 (0.22)	0.40 (0.23)	0.82 (0.30)
Northern/Western/Central Europe		20.03 (0.85)^+^	2.93 (2.19)	1.80 (1.14)
Remaining countries		1.38 (0.69)	2.40 (1.99)	1.43 (1.05)
**Citizenship**				
German and foreign		2.33 (0.57)***	1.61 (0.71)	3.29 (1.16)**
Foreign		1.02 (0.29)	1.02 (0.52)	0.86 (0.36)
Far-right voting z-standardized (FRV)			0.79 (0.07)**	
FRV × Older age			1.25 (0.15)^+^	
FRV × Gender			1.06 (0.14)	
**FRV × Birthplace**				
Southern/Eastern Europe			0.64 (0.33)	
Asia			0.41 (0.33)	
Northern/Western/Central Europe			0.59 (0.54)	
Remaining countries			1.56 (1.97)	
**FRV × Citizenship**				
German and foreign			1.56 (0.88)	
Foreign			1.25 (0.84)	
**School education**				
Secondary school				0.99 (0.17)
Upper secondary school				1.12 (0.25)
High school				1.00 (0.23)
Vocational training				
Vocational school				1.92 (0.39)**
Technical school				1.99 (0.31)***
Advanced technical school				1.95 (0.43)**
University				2.84 (0.68)***
Income z-standardized				1.51 (0.11)***
Children (N°)				0.90 (0.06)

	**Random effects (variances)**			

**Level 2 (company)**				
Number of level 2 units	1413	1413	594	992
Intercept	0.23 (0.08)	0.21 (0.08)	0.29 (0.12)	0.27 (0.16)

*Standard errors are in parentheses. Income is z-standardized.*

*FRV = far-right voting z-standardized.*

***p < 0.001; **p < 0.01; *p < 0.05; ^+^*p* < 0.10.

**TABLE 4:** Mixed multilevel analyses for job offers with fixed effects and random intercepts for company as second level.

Parameter	Model 1 (*n* = 2356)	Model 2 (*n* = 2356)	Model 3 (*n* = 1296)	Model 4 (*n* = 1250)
	
	Fixed effects (odds ratios)			
Intercept	1.13 (0.05)**	1.23 (0.08)**	1.23 (0.11)*	1.23 (0.22)
**Level 1 (employee)**				
Older age		1.06 (0.09)	0.96 (0.11)	1.07 (0.15)
Gender		0.68 (0.07)***	0.72 (0.10)*	0.77 (0.12)^+^
**Birthplace**				
Southern/Eastern Europe		0.93 (0.22)	0.46 (0.22)^+^	1.14 (0.37)
Asia		0.70 (0.22)	0.59 (0.33)	0.46 (0.19)^+^
Northern/Western/Central Europe		0.80 (0.39)	0.33 (0.31)	0.30 (0.22)^+^
Remaining countries		0.50 (0.28)	2.71 (4.18)	0.20 (0.18)^+^
**Citizenship**				
German and foreign		1.20 (0.31)	1.36 (0.77)	1.20 (0.45)
Foreign		1.19 (0.38)	3.43 (2.37)^+^	1.40 (0.62)
Far-right voting z-standardized (FRV)			0.91 (0.08)	
FRV × Older age			1.13 (0.14)	
FRV × Gender			0.91 (0.12)	
FRV × Birthplace				
Southern/Eastern Europe			0.43 (0.28)	
Asia			0.61 (0.46)	
Northern/Western/Central Europe			0.54 (0.81)	
Remaining countries			10.17 (19.32)	
**FRV × Citizenship**				
German and foreign			0.96 (0.79)	
Foreign			7.43 (7.32)*	
**School education**				
Secondary school				0.95 (0.17)
Upper secondary school				1.14 (0.26)
High school				1.06 (0.24)
**Vocational training**				
Vocational school				0.89 (0.19)
Technical school				1.04 (0.16)
Advanced technical school				1.19 (0.27)
University				0.81 (0.19)
Income z-standardized				0.99 (0.06)
Children (N°)				1.02 (0.07)

	**Random effects (variances)**			

**Level 2 (company)**				
Number of level 2 units	1109	1109	508	745
Intercept	0.05 (0.08)	0.07 (0.08)	0.12 (0.11)	0.00 (–)

*Standard errors are in parentheses. Income (net) is z-standardized.*

*FRV = far-right voting z-standardized.*

***p < 0.001; **p < 0.01; *p < 0.05; ^+^*p* < 0.10.

We started with null models to estimate what amount of variance can be explained by nesting participants in companies. In null models, differences between companies (L2V) explained about 6.50% (ICC = 0.065, *SE* = 0.022, 95% CI[0.033; 0.124]) of the overall variance in active sourcing (DV1) and about 1.62% (ICC = 0.016, *SE* = 0.023, 95% CI[0.001; 0.216]) of the overall variance in job offers (DV2).

In the next step, we calculated separate mixed effects logistic models, predicting active sourcing (DV1) and job offers (DV2) from age, gender, country of birth, and citizenship. We allowed the intercept to vary across different companies, our level-two variable. Note, however, that our IVs are categorical and that we did not center them.

**FIGURE 3:**
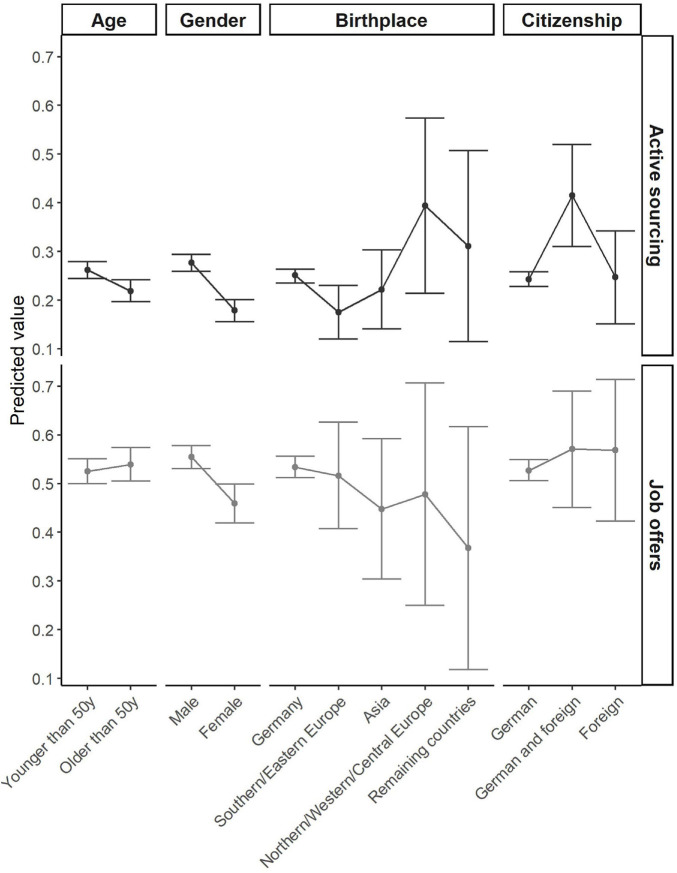
Predicted values for the two dependent variables, active sourcing (DV1) and job offers (DV2). The first row of boxes refers to active sourcing, the second row to job offers. The dots represent the predicted values, the angled lines represent the 95% confidence interval. The reference categories are younger than 50 years for age, male for gender, Germany for birthplace, and German for citizenship.

Figure 3 shows the predicted values of being actively approached or getting a job offer for the level-one predictors.

We found significant effects of gender, age, and migration background for active sourcing. The odds ratios of female employees being actively approached were significantly lower than for male employees (*OR* = 0.555, *SE* = 0.051, *p* < 0.001). This showed that female employees had a 45% lower chance of being approached by a recruiter. In addition to this gender effect, we found a significant age effect (*OR* = 0.783, *SE* = 0.066, *p* = 0.004) for active sourcing. Older employees had a 22% lower chance of being actively approached by recruiters than younger employees.

For ethnic minorities, we found mixed evidence with regard to active sourcing. People who were born in Southern/Eastern Europe were approached significantly less often than people who were born in Germany (*OR* = 0.620, *SE* = 0.131, *p* = 0.024), while there was no significant main effect for people born in Asia. If we instead looked at foreign citizenship, we found that people with dual German and foreign citizenship were even at an advantage. They had a significant higher chance of being actively approached than people who have German citizenship only (*OR* = 2.334, *SE* = 0.565, *p* < 0.001). However, there was no effect for people with foreign citizenship only.

Concerning the second DV, job offers, we only found a significant effect for gender (*OR* = 0.691, *SE* = 0.065, *p* < 0.001). Female employees had a significant 31% lower chance of getting job offers from another company than male employees. All other effects for marginalized social groups were not significant at *p* < 0.05 or did not show any trend at *p* < 0.10.

We did not find any interaction for gender, age, and migration background, either operationalized via country of birth or via citizenship. However, we had to exclude the category of remaining countries due to collinearity to compare models with and without interactions (Likelihood ratio tests for the inclusion of interactions for active search: LRchi2(16) = 16.06, prob > chi2 = 0.449; for job offers: LRchi2(16) = 11.37, prob > chi2 = 0.786).

**FIGURE 4:**
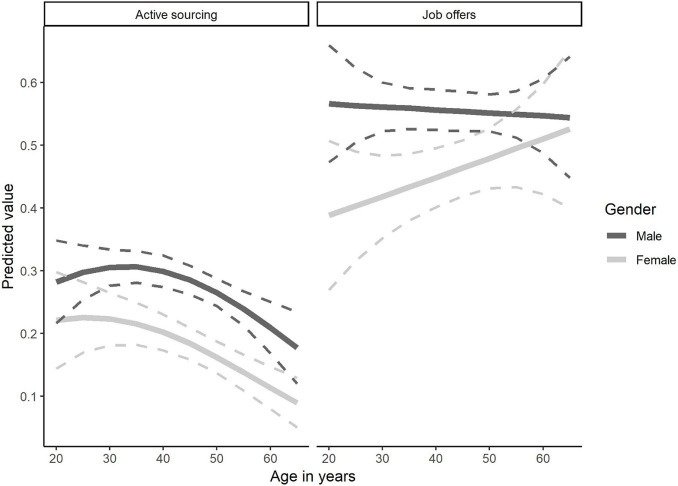
Predicted values for age and gender. The left side refers to active sourcing, the right side to job offers. The dark lines represent the effects for male employees, the lighter lines the effects for female employees. Solid lines represent the predicted values over the ages. The dashed lines indicate the 95% confidence interval.

While migration effects were ambivalent, gender and age effects were more prevalent in our models. Therefore, we calculated models with age as a continuous variable and added squared terms for age and interactions of age and gender. Gender effects were significant for both DVs. The squared age term was not significant for both DVs but showed a trend for active sourcing (*OR* = 0.999, *SE* < 0.001, *p* = 0.058). The interaction of gender and age was not significant for both DVs. Figure 4 shows the predicted values for male and female employees in different age groups for active sourcing and job offers.

#### Interaction With Regional Far-Right Voting

In the third step, we looked into interaction effects with far-right voting. We first added the share of the federal election’s (2013) far-right voting to our Model 2 and second, its interactions with the IVs—age, gender, country of birth, and citizenship. We used z-standardization for far-right voting to improve the possibilities for interpretation of interactions.

When we added far-right voting without interactions, we found a significant main effect for far-right voting on active sourcing (*OR* = 0.861, *SE* = 0.053, *p* = 0.017). It shows that in areas with one standard deviation more far-right voting, all employees had 14% less chance of being actively sourced. There was still a negative main effect for female employees. All other effects were not significant. However, this might also be a selection effect. Age and ethnic effects already disappeared in the reduced sample before we added far-right voting.

When we included interactions of IVs and far-right voting, the effect for far-right voting was even larger (*OR* = 0.785, *SE* = 0.068, *p* = 0.005). We did not find any significant interactions with far-right voting. However, we found a trend for a positive interaction for older people and far-right voting. Older people tend to be more actively sourced in districts with higher far-right voting (*OR* = 1.253, *SE* = 0.149, *p* = 0.058). This might be an artifact, however. There are more older people living in East Germany. At the same time, East Germany has numerous regions with a high share of far-right voting.

Concerning the DV2, job offers, we found no main effect for far-right voting when we included far-right voting without interactions. The negative effect for female employees was similar regardless whether interactions or just the main effect of far-right voting were included. We found one significant interaction with people who have foreign citizenship only (*OR* = 7.431, *SE* = 7.320, *p* = 0.042). However, due to the large standard error, we are hesitant to interpret this effect. We found two trends, one negative trend for people born in Southern/Eastern Europe (*OR* = 0.456, *SE* = 0.216, *p* = 0.098), and a positive trend for people who have foreign citizenship only (*OR* = 3.432, *SE* = 2.372, *p* = 0.075) when far-right voting and its interactions terms were included. The trend for Southern/Eastern Europe emerged only when all interactions were included simultaneously, however. The other trend for foreign citizenship has a large standard error. Therefore, we suggest caution in interpreting both trends.

#### Robustness Check

In a last step, we added several control variables to our model from step two to check the robustness of our effects. We controlled for level of school completion, vocational training, net income (z-standardized), and number of younger children in the household at the employee level. Originally, we also wanted to add far-right voting as a control variable, but this would have reduced our sample to less than 1,200 people for active sourcing and less than 700 people for job offers.

We first calculated our model using the reduced sample but without including the control variables themselves. In this reduced sample for active sourcing, we found no effect for age but a negative effect for gender (*OR* = 0.464, *SE* = 0.065, *p* < 0.001), a positive effect for people with dual citizenship (*OR* = 2.960, *SE* = 1.025, *p* = 0.002), and a negative trend for people born in Southern/Eastern Europe (*OR* = 0.603, *SE* = 0.176, *p* = 0.082).

The main effects for gender and for people with dual citizenship were still significant with controls at the employee level. Additionally, we found a negative effect for older people when control variables were included (*OR* = 0.637, *SE* = 0.091, *p* = 0.002). This might be a suppressor effect, which is difficult to interpret. Effects for income and vocational training were significant, but not for children or school education.

A multilevel analysis for job offers (DV2) with the reduced sample but without control variables showed negative effects for gender (*OR* = 0.754, *SE* = 0.106, *p* = 0.045) and for people who were born in Asia (*OR* = 0.438, *SE* = 0.176, *p* = 0.040). When we included the control variables on the employee level, the main effects for gender and people who were born in Asia were not significant but showed a trend. The question of whether the effects lacked robustness on job offers or whether the effects were mediated by the control variables is still an open one.

**FIGURE 5:**
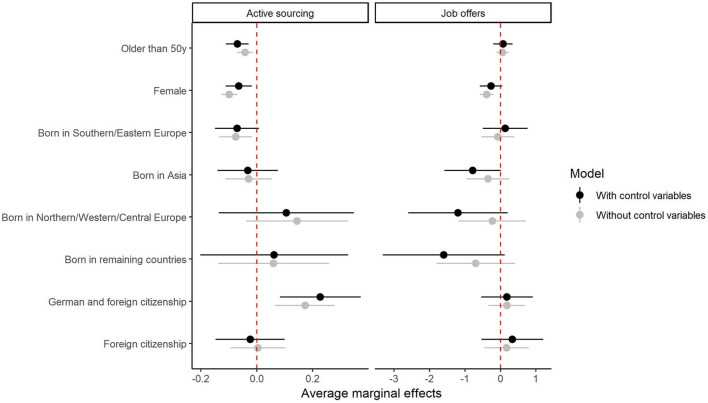
Average marginal effects for the two dependent variables, active sourcing and job offers. The first column refers to active sourcing, the second column to job offers. The reference categories are younger than 50 years, male, born in Germany, and German citizenship. The average marginal effects are represented by dots, the 95% confidence interval is represented by the horizontal lines at each point. Dark lines represent the effects with control variables, light lines the effects without control variables. The vertical dashed line at zero represents the threshold for positive and negative effects.

Figure 5 shows the average marginal effects for both DVs calculated with and without control variables. Average marginal effects were calculated using marginal predicted means for active sourcing and linear regression for job offers.

### The Discussion of Quantitative Results

We found evidence that female candidates, older candidates, and candidates who were born in Southern/Eastern Europe were actively sourced less often. Female candidates were also offered job offers less often. Our mixed multilevel models showed that the company has a substantial impact on the likelihood of being actively sourced.

#### Effects for Marginalized Groups in Active Sourcing

We found significant gender effects in our analyses. Female employees were actively sourced less often and were offered job offers less often from other companies. This effect was robust when conducting multilevel analyses and when controlling for school education, vocational training, income, and number of young children in the household. We consider this initial evidence of potential unequal treatment of female employees in the current recruiting environment. Companies use more active sourcing strategies, especially for high management and well-paid jobs. We regard this as a risk that gender inequality in labor market will not only continue but also increase with the enhanced use of active sourcing strategies. Researchers might take this into consideration when conducting studies on hiring discrimination.

We also found that older employees were actively sourced less often, while they were not at any disadvantage when it came to job offers. However, this effect was not very robust in multilevel analyses and in analyses controlling for school education, vocational training, income, and the number of young children in the household. Further research is required to examine whether or not active sourcing strategies systematically discriminate against older people.

We found mixed effects for potential discrimination based on ethnic origin. People who were born outside Germany tended to be sourced less often; but this effect was significant only for people born in Southern/Eastern Europe. In addition, we were not able to control for language barriers. At the same time, people with dual citizenship were sourced more often than people who only had German citizenship. However, information on employees’ ethnic origin in the IAB data is very rough and makes it impossible to identify the offspring of migrants. Our sample was therefore able to identify the first generation but not the second and third generations. It was not possible to operationalize migration background in the same way as in the federal statistics, where at least one parent needs to be born in a foreign country (Statistik der Bundesagentur für Arbeit, 2020). This weakness of the data also had consequences for the reference category. Ideally, the reference category is all Germans without foreign roots. Our reference includes any person in Germany without foreign citizenship and a German birthplace. This made it even more difficult to find significant effects.

#### The Influence of Far-Right Voting

When we included far-right voting, we did find a main effect for far-right voting but no significant interactions with the marginalized groups as we assumed. One reason might be that we were able to add election data for the regional district code—which is a rather large regional unit—but not for the constituency. We found a main effect for female but not for older people. The interactions of older people with far-right voting showed a positive trend that is in line with our hypotheses. But the interactions with other social groups did not reach significance. Far-right voting apparently has no moderating effect on active sourcing or job offers to members of marginalized groups, but it does have a negative main effect on active sourcing. This suggests lower use of modern recruitment strategies in regions with a more right-wing normative climate. Note, however, that this is an ad-hoc explanation. We hope that this will be addressed in future research.

## General Discussion, Implications, and Limitations

Our research question was to evaluate to what extent modern recruitment is affected by discrimination against marginalized group members. We conducted a mixed-method study with two separate empirical data sources. The combination of qualitative interviews with quantitative analyses provides a better understanding of the discriminatory mechanisms at work in modern recruitment. We conducted eight semi-structural qualitative interviews and explored the contemporary recruitment process and the potential for implicit or explicit discriminatory decisions. Subsequently, we conducted mixed multilevel analyses to find out whether or not employees who belong to marginalized groups were less often actively approached and less often offered a new job.

Content analyses of our qualitative interviews identified some aspects in the modern recruitment process that might bear a risk for discrimination against these candidates. This risk is not related to implicit or uncontrolled processes but is mostly motivated in an explicit manner. We consider *active sourcing* together with *recruitment assignment* to pose a major risk of explicit or controlled discrimination. In contrast to previous research on traditional recruitment, our findings suggest that time pressure is not a major problem in the modern recruitment process. The initial idea that recruitment is affected by implicit discrimination does not appear to be confirmed. In fact, it seems that screening, which is potentially affected by implicit discrimination, does not play a major role in modern recruitment. This is an important insight, which could only be obtained because qualitative interviews were not restricted to predefined questions. It is a valuable advantage of the qualitative interviews that recruiters had the opportunity to describe in their own words how they conducted the recruitment process. If we had followed standardized interview guidelines, it might not have become clear that screening is unimportant in modern recruitment. Our analysis revealed that personnel selection is partly based on personal preferences and not strictly on best qualification. We identified three sources for this potential discrimination: the recruiters’ own prejudices, the managers’ expressed preferences, and the assumed company’s or managers’ preferences.

In our quantitative analyses, we were able to test our hypotheses in a representative sample. We found further evidence that employees who belong to marginalized groups were actively approached less often in active sourcing attempts. While it is too early to claim ethnic discrimination, we found evidence that female and elderly people might indeed be discriminated against in active sourcing. Female and older employees as well as most people who were born outside Germany were less often actively approached. Additionally, female employees were offered job offers less often than male employees. These gender effects also reached significance when controlling for school education, vocational training, income, and numbers of children. It is important to note that in the quantitative analyses, we were not able to ask about the motivational underpinnings of the behavior. We focused instead on evidence of discrimination in active sourcing. We used the results of the interviews to identify this approach as the main one potentially affected by discrimination. The discrimination that is driven by this mechanism might therefore be explicit and controlled. We found no significant interaction of our predictors and far-right voting. However, we found a significant negative main effect for far-right voting. This ties back to our interview study, in which recruiters reported that they take into account the region where their client is based. We consider the latter further evidence that the third source of discrimination—the assumed preferences of companies’ managers—might have an impact on recruitment decisions. Assumed preferences or meta-stereotyping might also be important when exploring the potential discrimination in contemporary recruitment, among individual recruiters’ and managers’ prejudices. Some strands of psychological research point to this process. Research about *meta-cognition* (Greifeneder and Schwarz, 2014) or *social norms* (Cialdini and Trost, 1998) show how individuals assume preferences from other people and base their behavior on these assumptions. The concept of *shared mental models* (Mathieu et al., 2000) describe how members of a team understand each other.

All these models are helpful to explore this effect. However, there is a lack of recruitment research taking this into account. While much research has been conducted on traditional recruitment, less has been done on recruitment trends such as active sourcing and recruitment assignment. More research needs to be conducted to apply models of meta-stereotyping in recruitment.

### Undermining the General Equal Treatment Act

We think that the recruitment trends *active sourcing* and *recruitment assignment* might undermine current German legislation that aims to protect future employees from discrimination. The GETA was passed by the German Bundestag, Germany’s federal parliament, in 2006, with the aim of preventing or eliminating discrimination “on the grounds of race or ethnic origin, gender, religion or belief, disability, age or sexual orientation” (Bundestag, 2006).

However, our results show that employee protection legislation might be insufficient to protect future employees in modern recruitment environments. The first problem relates to equal treatment requirements. Employers increasingly hire external agencies to do their recruiting, and recruiters from external agencies feel much more bound by the companies’ (hidden) preferences than by GETA. As a consequence, companies can avoid candidates from groups they dislike without getting into conflict with the law. Companies’ official documents—for instance, job advertisements or business profiles—would not arouse any suspicion among job candidates. Companies put a great deal of effort into the wording of these official documents so that everything that is published conforms to GETA.

*(16) What important aspects are not covered in job advertisements? Because job advertisements are all consistent with GETA, but of course positions are often filled based on different criteria. (i7)*

The second problem is related to active sourcing activities in modern recruitment processes. Here—unlike in classic recruitment process—job candidates do not apply for jobs. The recruiters select the candidate who they are interested in. Candidates would not know if they were being actively sourced less often by recruiters. When diverse job candidates do not know about the discriminatory selection, they cannot lodge a complaint. Therefore, it is almost impossible to accuse anybody of discrimination in active sourcing.

These problems illustrate how recruitment trends like *active sourcing* and *recruitment assignment* to external agencies might undermine the intended purpose of GETA.

### Weakening of Works Councils’ Impacts on the Modern Recruitment Process

Works councils in Germany are allowed to demand more diversity among shortlisted job candidates if they feel a shortlist has been discriminatory (Bundestag, 1972). One might state that a strong works council is able to prevent hiring discrimination. We argue that works councils’ impacts are limited to classic recruitment and in-house recruitment processes, whereas they might lose their anti-discriminatory control in modern recruitment processes.

When a private agency is contracted to complete the recruitment process, works councils will only see the shortlisted candidates. The same holds true for active sourcing. When recruiters use active channels, there will be no official list of candidates before shortlisting. Often, external recruiters will not present more than three final candidates in their shortlist. This makes it impossible for works councils to prevent discrimination. Off the record, an interviewed recruiter put it like this: When the manager has a preference for male candidates, then the recruiter will pretend that—coincidentally—only male candidates were interested.

Additionally, the company does not necessarily have information on how the external recruiter has conducted the recruitment and which candidates the recruiter has already excluded. The works council, on the other hand, does not have the right to demand information from outside firms—which is the case with external recruitment agencies. Therefore, the works council might not be able to protect marginalized candidates from discrimination in recruitment processes that have been assigned to recruitment agencies.

### Limitations

Our mixed-method study has several limitations.

#### Limitations in the Qualitative Interview Study

Our qualitative interviews were selected through snowball sampling. This bears the risk of a certain selectivity. Therefore, the content might not be representative of the entire labor market in Germany. We welcome further research that overcomes this selectivity with a better sampling method.

It was difficult to find in-house recruiters for our expert interviews. Only two in-house recruiters took part. Our findings may therefore reflect the drivers of discrimination among recruiters in recruitment agencies better than among in-house recruiters.

Apparently, the modern recruitment process reflects the current labor market situation in Germany. For the period of time under study, the German labor market was very tight. This meant that employers had difficulties filling job openings, especially in fields where there were labor shortages. This might motivate companies to use more active sourcing tools or assign recruitment to external recruitment agencies. However, if the labor market changes, the recruitment process will change in the years that follow. Accordingly, our conclusions need to be assessed in future recruitment landscapes.

#### Selectivity and Small Group Sizes in the Panel Study

One problem of our panel study was that our sample might be selective. Our main DVs were conditional on the people’s turnover intention. Only employees who answered that they were thinking about changing jobs were asked whether companies had approached them and whether they had received job offers. This bears the risk that our sample includes many people who were unhappy or even unsuccessful in their job. Moreover, our interviews with recruiters suggest that recruiters do not wait until a person has a turnover intention. They try to convince potential candidates by making attractive job offers. We consider this a substantial potential selection bias of our study.

A further limitation is that our sample included very few people of non-German origin. This might be a reason why we did not find overall effects for people who were born outside Germany. We did report the effects for reasons of completeness, but we urge caution in interpreting them. Further research is needed to explore these specific effects in more detail.

#### Missing Data

A strong limitation of our quantitative analyses is the high share of missing data in the variables of interest. We “lost” almost half of the sample when merging data from employee and employer surveys. This becomes particularly evident when controlling for employee characteristics in step four. This is why we chose the control variables very carefully and included only those with relatively little missing data. This made it very difficult to confirm significant differences and made the analyses prone to selection biases. The high share of missing data was also the reason why it was not possible to control for local characteristics, such as unemployment ratios and the percentage of foreigners. Both control variables would have been very valuable in checking the robustness of the effects, but these variables are only available in other data sources and matching more data sources would have further reduced our sample. In this case, the balance between need for controls and research interest is a delicate one. To the best of our knowledge, however, there is no better data available for the German labor market than the LPP. The LPP is an add-on survey study of the establishment panel—the official panel study used by the German government to survey labor market trends and needs. This means that the sample is selected in a very thorough process and allows for generalization of effects. What we found interesting was that, although the data are very limited, we found meaningful initial evidence that active sourcing might be affected by discrimination against women and—to a certain extent—older candidates. We hope that future research will follow up on this finding with a better data source.

#### Unequal Treatment Due to Qualification Differences

The last point refers to the interpretation of the effects. We are not able to decide whether this unequal treatment reflects discrimination or differences in qualification. Our interviews show that recruiters use active sourcing in jobs with high labor shortages and in top management positions. Female employees are underrepresented in many fields with labor shortages, such as IT and engineering. We were not able to control for the jobs that employees held in our models. Therefore, we cannot test for discrimination but only describe differences in averages. Nevertheless, our models with control variables for income and vocational training give further evidence that this unequal treatment might be discriminatory, at least against female employees. Controlling for income and vocational training rule out the explanation that women are often employed in low-wage and low-skilled occupations and are therefore sourced less often than men. However, further research is needed to explore in more detail whether candidates from marginalized groups are being discriminated against in modern recruitment.

### Implications: Training Companies to Value Diversity

Our findings about potential discrimination in modern recruitment have significant practical implications. Many companies have labor shortages and need to hire new talent. Our mixed-method study suggests that recruiters sometimes assume preferences and might sort out employees who are indeed qualified but who belong to marginalized groups. Companies are well advised to explicitly highlight the value they place on diverse employees when they meet with recruiters. This might prevent recruiters from assuming preferences and sorting out qualified candidates. Sometimes, assumed preferences might dovetail with managers’ expectations. We think it is important that companies understand that stereotypical preferences are not helpful in finding qualified personnel. The world of work is becoming more and more globalized and so is the world of recruitment. In our view, companies that understand the importance of fairness and that value diversity will profit from the so-called “war for talent.” In the end, it is not only a question of individual diversity but also one of how diversity can be achieved on a team and company level. Fairness is not just a moral question but also a pull factor for job applicants. In addition, studies have revealed that diverse teams seem to be more creative in finding solutions to problems. Many organizations already invest significant resources in diversity management. Knowing more about the discriminatory mechanisms at work in modern recruitment strategies might help them to become more effective in their diversity management practices. To achieve more diverse and more equitable workplaces, a clear focus should be placed on increasing fairness in all recruitment processes in the years to come.

## Data Availability Statement

The qualitative datasets presented in this article are not readily available because interviewees were guaranteed that we do not share the interview transcripts. Requests to access the qualitative datasets should be directed to EK, esther.kroll@wzb.eu.

## Ethics Statement

The patients/participants provided their written informed consent to participate in the qualitative study. This approach allows us to self-certify ethical approval for the qualitative study at the WZB Berlin Social Science Center in March 2017.

## Author Contributions

All authors made valuable contributions to the manuscript’s structure and content, and contributed to the article and approved the submitted version.

## Conflict of Interest

The authors declare that the research was conducted in the absence of any commercial or financial relationships that could be construed as a potential conflict of interest.

## Publisher’s Note

All claims expressed in this article are solely those of the authors and do not necessarily represent those of their affiliated organizations, or those of the publisher, the editors and the reviewers. Any product that may be evaluated in this article, or claim that may be made by its manufacturer, is not guaranteed or endorsed by the publisher.

## Supplementary Material

Supplementary Table 1 Methods of the qualitative interview study.

Supplementary Table 2 Original German interview quotations.

Supplementary Data Sheet 1 STATA output of the calculations.
