# Supplementary material for: The Discriminatory Potential of Modern Recruitment Trends—A Mixed-Method Study From Germany
Source: Front Psychol. 2021 Oct 25;12:634376. doi: 10.3389/fpsyg.2021.634376 (PMC8573412; doi:10.3389/fpsyg.2021.634376)
Supplement: Supplementary Table 1 — Methods of the qualitative interview study. [file Table_1.DOCX]

# *Methods of the qualitative interview study*

# Expert interviews

Most HR staff members have more responsibilities than recruitment alone, for instance, personnel development or administrative tasks. We therefore interviewed recruiters who focused solely on recruitment. We use the term “recruiter” to describe all of the interviewees’ professions. These recruiters are able to explain how recruitment trends such as the use of professional social network sites (SNS), active sourcing, and recruitment assignment to external agencies play a role in the recruitment process. They are also able to reflect on current challenges in recruitment and compare in-house and external recruitment processes. Additionally, recruiters often have unique insights into companies’ hidden agendas. They know much more about the preferred profile of candidates than can be determined by looking at public documents such as job advertisements. If a company’s management has discriminatory preferences, recruiters are often the only ones aside from management who have access to this confidential information. Professional recruiters are therefore able to describe official and unofficial strategies to find and select candidates.

# The selection of interviewees

In the selection of our interviewees, we searched for a variety of recruiters to get a comprehensive picture of modern recruitment processes. We selected our interviewees according to the following rules:

- The interviewee needed to be a recruiter, personal consultant, or HR staff member with the primary task of sourcing and selecting future employees.
- We selected only interviewees who used professional SNS platforms in their recruitment process.
- In-house recruiters and recruiters from external agencies both qualified as long as their primary task was to recruit future employees.
- We selected recruiters from various recruitment areas, for example, focusing on different positions (e.g., executive search and search for skilled employees) and different labor market situations (e.g., demand and supply: with and without labor shortage).
- Interviewees were only allowed to take part in the interview after signing the informed consent form.

We realized that the recruiters’ answers were relatively homogeneous. After the first interviews, no further topics arose during the sessions. Therefore, we stopped sampling after eight interviews.

# Interview guidelines

In line with our research interest, we developed 34 interview questions and used them as our interview guidelines. The interview guidelines included questions on working conditions and operational procedures as well as questions on search goals, because both are important to explore when analyzing the discriminatory potential of recruitment trends. If the working conditions imply high time pressure and cognitive load, this will increase the potential of implicit discrimination. If the recruiters’ motivation is to exclude candidates with certain personal characteristics, this will increase the potential of explicit discrimination.

The interview guidelines aimed at structuring the interview and ensuring that that all topics of interest would be discussed in the interview. However, it is important that expert interviews are flexible, so that the individual has the chance to express his or her unique point of view. Therefore, we used the guidelines that we developed as a supportive tool rather than as a strict rule. During the interviews, the order of questions varied according to interview partners’ interests. We also added new topics that were raised by interviewees.

We used the first interview as a pre-test of the guidelines, which we adapted thereafter. This first interviewee was an in-house recruiter who had formally worked under contract as an external recruiter one year before. We realized that in-house and external recruitment definitions might vary among interviewees. In the revised interview guidelines, we added questions 4 and 5. These questions are in italics.

The revised interview guidelines read as follows. We include an English translation in parentheses below each question:

1. Es wird im Moment viel darüber diskutiert, dass sich die Arbeitswelt der Zukunft verändert und weiter verändern wird. Was sind Ihrer Meinung nach die größten Herausforderungen an die Personalgewinnung der Zukunft?

[There is a lot discussion right now about how the working world is changing and will continue to change in the future. What is the biggest challenge, in your opinion, for recruiting in the future?]

1. Wenn ich es richtig verstanden habe, wird in Ihrem Unternehmen Personal für andere Firmen gesucht. Unter welchem Begriff lassen sich Personaldienstleister wie Ihr Unternehmen zusammenfassen?

[If I understood correctly, your company searches for personnel for other companies. What term do you use to describe personnel agencies like your company?]

1. Wie ist das, wenn jemand auf der anderen Seite als Recruiter arbeitet. Gibt es verschiedene Jobbezeichnungen für dieselbe Tätigkeit?

[What about when someone works as a recruiter: Are there different job names for the same task?]

1. *Welche Gründe gibt es für Unternehmen, die Personalsuche „out zu sourcen“?*

*[What are the reasons why companies outsource the recruitment process?]*

1. *Worin unterscheidet sich Ihrer Meinung eine Inhouse-Personalsuche zu einer out gesourcten Personalsuche?*

*[What do you think the difference is between an in-house and an outsourced recruitment process?]*

1. Mich interessiert die Zusammenarbeit zwischen Ihnen und dem Auftraggeber. Wie kommt eine Kooperation zwischen Ihrer Firma und dem Auftraggeber zustande?

[I am interested in how you and the client work together. How does the partnership come about between your company and the company that enlists you to do its recruiting?]

1. Welche Information zum gesuchten Bewerber wird Ihnen vor der Suche zur Verfügung gestellt? Gibt es Vorgespräche mit den Auftraggebern? Was wird in der Regel in Vorgesprächen miteinander besprochen/ aufeinander abgestimmt?

[What information do you receive about preferred candidates before beginning the search process? Are there meetings with the client? What do you discuss or agree on in these preliminary meetings?]

1. Wer bestimmt die Kanäle, über die Personal gesucht wird?

[Who decides which channels will be used to search for personnel?]

1. Als nächstes würde ich gerne einen Überblick über Ihren Arbeitsalltag bekommen. Wie sieht ein typischer Arbeitsalltag eines Recruiter aus?

[Next, I would like to get an overview of your day-to-day work. What does a recruiter’s typical working day look like?]

1. Welche verschiedenen Aufgaben werden an einen Recruiter gestellt?

- Aktive Suche
- Vorauswahl
- Kontaktaufnahme mit Bewerbern
- Besprechung mit Auftraggeber

[What tasks does a recruiter have?

- Active sourcing
- Creating a shortlist
- Contacting job candidates
- Meeting with the client]

1. Wie viel Zeit benötigen die Aufgaben am Tag durchschnittlich?

- Aktive Suche
- Vorauswahl
- Kontaktaufnahme mit Bewerbern
- Besprechung mit Auftraggeber

[How much time do you need for the different tasks each day?

- Active sourcing
- Creating a shortlist
- Contacting job candidates
- Meeting with the client]

1. Wie viele Stellen besetzen Sie durchschnittlich pro Tag /pro Woche?

[How many job openings do you fill each day/each week on average?]

1. Zur aktiven Personalsuche greifen Sie und Ihre Kollegen auf berufliche Plattformen zurück. Ich habe einige Fragen zur konkreten Nutzung der Plattformen:

Welche Vorteile sehen Sie als Anwender in beruflichen Online-Plattformen?

[You and your colleagues use professional SNS for active sourcing. I have several questions about the concrete use of SNS: What advantages do you, as a user, see in professional SNS?]

1. Gibt es neben der Personalsuche weitere Anwendungsbereiche von Karrierenetzwerken?

[Are there other uses of SNS, besides in searching for personnel?]

1. Wie sieht die Personalsuche mittels beruflicher Plattformen in der Regel aus?

[What does the search for personnel usually look like when you use professional SNS?]

1. Wenn man die Personalsuche an einem Beispiel durchspielt: Wie gehen Sie vor um eine Vorauswahl der Kandidaten zu treffen?

[If we went through a personnel search as an example: How would you create a shortlist of candidates?]

1. Gibt es Begriffe, die Sie häufig bei der Suche nach geeigneten Kandidaten einsetzen?

[Are there certain keywords that you often use to search for suitable candidates?]

1. Was wird Ihnen nach Ihrer Suchanfrage als erstes angezeigt?

[What is the first thing that appears when you do a search?]

1. Worauf achten Sie bei einem Bewerberprofil am meisten?

[What is most important to you in a candidate’s profile?]

1. Was fällt an einem Bewerberprofil als erstes auf?

[What is the first thing you notice about a candidate’s profile?]

1. Inwiefern wird das Netzwerk eines Kandidaten bei der Personalsuche mit beachtet?

[To what extent do you take the candidate’s network into account in your personnel search?]

1. Was macht ein gutes Bewerberprofil aus?

[What does a good candidate’s profile look like?]

1. Welche Bewerberprofile werden kategorisch aussortiert bzw. übergangen?

[What profiles do you sort out or skip over categorically?]

1. Wie wichtig glauben Sie ist ein ansprechendes Bewerberfoto?

[How important is a good job application photograph in your opinion?]

1. Sie haben ja schon einen reichen Erfahrungsschatz. Können Sie Kriterien ausmachen, die Sie zur Einschätzung eines Bewerbers heranziehen, die sich aber nicht direkt auf die Ausbildung oder den beruflichen Werdegang beziehen?

[You have extensive experience. Are there any criteria that you take into account when assessing a candidate that do not relate directly to education or professional background?]

1. Nachdem Sie Ihre Suchanfrage gestellt haben, wie viele Profile schauen Sie sich ca. an?

[After doing a search, approximately how many profiles do you look at?]

1. Können Sie einschätzen, wie lange Sie auf einem Profil durchschnittlich verweilen?

[Can you estimate how much time you spend on each profile on average?]

1. Wie viele Bewerber sichten Sie durchschnittlich, bevor Sie Ihre Kandidaten für die Vorauswahl gefunden haben?

[How many candidates do you look at on average before you find a candidate for your shortlist?]

1. Wie viel Zeit verwenden Sie um sich einen Überblick über die Bewerberlage einer Position zu verschaffen? Gibt es einen Zeitrahmen, nach welchem Sie in der Regel eine Vorauswahl treffen?

[How much time do you spend to get an overview of the pool of candidates for a position? Is there any time frame in which you usually need to complete your shortlist?]

1. Wie viele Bewerber werden in die Vorauswahl in der Regel aufgenommen? Wie viele Kandidaten werden dem Auftraggeber präsentiert?

[How many candidates do you usually have in the shortlist? How many candidates do you present to the client?]

1. Haben Sie die Erfahrung gemacht, dass es eine bestimmte Präferenz für ein bestimmtes Alter eines Bewerbers, oder Geschlecht oder regionale Anbindung oder nationale Herkunft eines Bewerbers gibt?

[Have you found that there is a certain preference for a certain age, gender, or regional connection, or for the ethnic background of the candidate?]

1. Inwiefern werden Sie aufgefordert den Vorgaben/Präferenzen zu folgen?

[To what extent are you asked to follow the preferences?]

1. Wenn Sie eine Vorauswahl getroffen haben, was passiert im Anschluss?

[When you have made a shortlist, what happens in the next step?]

1. Wir haben viel über Karriereportale gesprochen. Gibt es aus Ihrer Sicht Alternativen zu beruflichen Plattformen, um effektiv Personal zu suchen?

[We have spoken a lot about professional SNS. Are there any alternatives to professional SNS in your opinion to search for personnel effectively?]

# Content analysis

We conducted a content analysis (Mayring & Fenzl 2014) to evaluate to what extent modern recruitment trends are affected by implicit or explicit discrimination. Additionally, we analyzed the structural features and the sources of discrimination in modern recruitment processes. First, we recorded interviews in audio form and fully transcribed them afterwards. Before we started to work with the transcripts, individuals’ and companies’ names were replaced by capital letters and numbers. We coded the interviews, first driven by theory and then by data. For the coding, we used four interview themes that we already had in our interview guidelines and added one topic upon examining the interview material. We discovered that recruiters spoke extensively about changing recruitment environments. We therefore added one code to the theme “current changes in recruitment”. Every code represented one research theme. The five major themes for the coding were: (1) current changes in recruitment, (2) structural features of the recruitment process, (3) the process of external recruitment and in­-house recruitment, (4) the use of professional SNS in the recruitment process, and (5) discriminatory recruitment behavior. We assigned colors to interview text blocks that were related to one of the research questions. Additionally, we wrote short memos on the codes, which summarized the text blocks. For the content analysis, we collected memos according to the themes and searched for consistent answers within the memos.

## The analysis of the modern recruitment process

To gain insight into modern recruitment procedures, we were interested in how recruiters usually go about recruiting. We assigned interview answers to Topic (2), structural features, where interviewees described the procedure of their recruitment process. Subsequently, we illustrated the recruitment process for various interviewees until we found a common structure. We then attempted to check whether recruiters considered the recruitment trends (professional SNS, active sourcing, and recruitment assignment) relevant and conducted a content analysis from the text blocks and memos assigned to Topic (1), current changes in recruitment. We assigned interview answers to this topic whenever recruiters described current trends and drivers in the recruitment environment. We analyzed how important a role our interviewees considered the recruitment trends to play in the current changes.

## The analysis of implicitly driven recruitment decisions

To evaluate the discriminatory potential of implicitly driven recruitment decisions, we analyzed whether conditions for automatic behavior are related to systematic rejections of minority group members.

First, we analyzed the implicit conditions of the recruitment trends in a content analysis with Topic (3), the process of external recruitment and in-house recruitment, and Topic (4), the use of professional SNS in the recruitment process. In Topic (4), we were especially interested in the time frame and the structural procedure when recruiters described profile screening. Here we looked in detail at whether recruiters described conditions that typically involve time pressure or a lack of structure in search procedures. We considered recruiters to be subject to time pressure either if they reported feeling rushed or if they reported making recruitment decisions within a very short time frame, i.e., within a few seconds. A procedure was considered unstructured if recruiters had no clear and explicit rules for evaluating candidates. This was the case, for instance, if recruiters described making their decisions based on their gut feelings.

Subsequently, we analyzed whether these implicitly driven recruitment decisions were related to a potential discrimination against minority candidates. We considered this evident if recruiters either described a potential exclusion or rejection of minority members, or if they described that their implicit recruitment decisions were not based strictly on the candidates’ qualities.

## The analysis of explicitly driven recruitment decisions

To evaluate the discriminatory potential of explicitly driven recruitment decisions, we analyzed whether conditions for controlled behavior are related to systematic rejections of minority group members.

We again conducted a content analysis with Topic (3), the process of external recruitment and in-house recruitment, and Topic (4), the use of professional SNS in the recruitment process. This time, we focused on recruitment assignment in Topic (3) and on active sourcing in Topic (4). We explored to what extent recruiters described being motivated to avoid mistakes in their pre-selections. Here, a mistake means that the client—usually the company’s hiring managers—reject the pre-selected candidate. We regarded this as the motivation to avoid failure that drives controlled behavior, according to Fazio (1990).

Subsequently, we analyzed whether these explicitly driven recruitment decisions are related to potential discrimination against minority candidates. In contrast to potential implicit discrimination, we considered this evident only if recruiters described the systematic exclusion or rejection of minority group members.

## The analysis of the sources of discrimination

If recruiters described discriminatory recruitment decisions against minority candidates, we aimed at analyzing where their discriminatory attitudes come from. We considered these attitudes potential sources of discrimination. First, we analyzed every text block in Topic (5), where recruiters described a systematic exclusion of a job candidate because of specific personal characteristics, for instance, because of the candidate’s ethnicity, gender, age, or ethnic origin. We conducted a content analysis to find out what drives the exclusion of those job candidates who are minority group members. We analyzed who (the recruiter or the companies’ managers) initiated the discriminatory decisions and how the decisions were made (independently of or dependent on others).
