# Supplementary material for: The Discriminatory Potential of Modern Recruitment Trends—A Mixed-Method Study From Germany
Source: Front Psychol. 2021 Oct 25;12:634376. doi: 10.3389/fpsyg.2021.634376 (PMC8573412; doi:10.3389/fpsyg.2021.634376)
Supplement: Supplementary Table 2 — Original German interview quotations. [file Table_2.DOCX]

**Original German interview quotations**

1. Ich habe auch Anfragen, da gebe ich wirklich nur die Stellenbezeichnung ein und Berlin und Brandenburg und ich habe null Treffer - also auch das gibt es. (i6)
2. Das Netzwerk spielt dahin gehend eine Rolle, dass ich Zugriff auf dieses Netzwerk habe und gucken kann: Wer bewegt sich in diesem Netzwerk? - Weil gute Leute – (das) ist eine ganz alte Weisheit - kennen nur gute Leute. (i2)
3. Hier sind alte Herren unterwegs, die nach wie vor ihre Notizbücher haben und ihre Netzwerke und das aktivieren. Also die Personalberatung im executive search - Bereich ist eine „good old company“. Eine ganz seniore, ein bisschen altbackene, in die Jahre gekommene, von persönlichen Profilen und von - ja - ganz besondere Netzwerkaktivitäten lebende Branche. (i3)
4. Wenn Sie wissen, der Kunde hat seinen Sitz in einer ganz bestimmten Stadt, können Sie sehr wohl aus guten Gründen mal überlegen: Will ich denn eigentlich hier die großartige Mobilitätsfrage stellen oder will ich das nicht? - Ob das denn so klug ist, ist eine gang andere Frage, aber es wäre eine Möglichkeit. (i3)
5. Natürlich, wenn ich bundesweit suche und einfach als Stichwort Elektroingenieur eingebe, dann sind es eher vierstellige Suchergebnisse. (i6)
6. Direktansprache heißt, die Leute vom Unternehmen direkt abzuwerben, im Unternehmen anzurufen. Das können wir quasi wegen Imageschaden einfach nicht machen. (i4)
7. Und wir arbeiten beispielsweise wie fast - oder wie die allermeisten Personalberater auf Erfolgsbasis. Das heißt wir werden nur dann honoriert, wenn wir die Stelle auch besetzen. Damit hat die Firma erstmal finanziell keine Belastung, wenn die halt Personalberater an Bord holt. (i6)
8. Wir machen unseren Job ja nicht richtig oder wir können es nicht erfüllen, wenn wir Kandidaten vorstellen und gar nicht wissen, dass die irgendwie an der gläsernen Wand abprallen. (i8)
9. Was z.B. auch ein großen Ausschlusskriterium bei mir ist- da muss man sagen, da gebe ich den Leuten vielleicht auch manchmal nicht die Chance, die sie verdienen. Aber wenn jemand eine größere Lücke drinnen hat, und es gibt keine Weiterbildung, nichts, dann fehlt mir - ich persönlich muss sagen, dann fehlt das Engagement. und die sind raus! Oder so Sabbatical drei Jahre. Das ist für mich wie ein rotes Tuch eigentlich. (i5)
10. *Bei indischen Bewerbern ist es auch häufig so, dass in Indien - das hat mir auch mein Fachbereich erzählt, auch IT-Fachbereich, die arbeiten ganz viel mit Dienstleistern in Indien, dass Loyalität zum Unternehmen ganz anders ist. Also wenn in Indien man ein paar Euro mehr in einem anderen Unternehmen bekommt, dann wechselt man. (i4)*
11. Und wir haben auch Unternehmen, also wir haben auch Personal oder auch Fachbereiche, die ganz klar sagen: Ich will nur eine Frau. oder: Ich will nur einen Mann haben. oder: Wir kriegen auch das Alter vorgegeben manchmal. (i7)
12. Es gibt oft Kunden, die sagen ganz klar: Ich habe da so einen Männerüberschuss, das ist so, das verroht so - es wär vielleicht schön, wenn mal wieder eine Frau ins Team reinkommt. - So als Beispiel, damit das Rülpsen aufhört oder damit die den Schreibtisch mal wieder sauber kriegen, wie auch immer. (i5)
13. Ich stelle z.B. die Frage: Darf es denn auch - muss es denn ein Mann sein?" - Ja. - "Ab welchem Alter darf der bei Ihnen nicht mehr rein?" - ja, das klingt sehr salopp die Frage. Und je salopper Sie es stellen, umso besser kriegen Sie dich auch beantwortet. Wir sind alle sehr sensibel in Sachen Antidiskriminierung. (i3)
14. Es gibt Firmen, da können Sie keinen Farbigen präsentieren. Und die Firma erklärt Ihnen steif und fest, dass es dazu rationale Gründe gäbe. Und Sie glauben das nicht. (i3)
15. Wenn ich jetzt für ein Unternehmen z.B. in Mecklenburg-Vorpommern suche, dann kannst du vielleicht nicht so multikulturell an die ganze Sache herangehen, weil da werde ich mehr „Neins“ bekommen als ein „Ja“. So, also, das heißt, ich gucke auch immer mal so regional bedingt, wo kommen die Unternehmen her und muss dann leider Gottes auch manchmal schon ein paar tolle Kandidaten ausschließen, die eigentlich passen könnten. (i5)
16. Was ist wichtig, was nicht in der Stellenausschreibung steht? - Weil die Stellenausschreibung sind ja alle AGG-konform, aber oft ist es natürlich auch so, dass Positionen mit einem anderen Hintergrund besetzt werden. (i7)
