# Supplementary material for: The Discriminatory Potential of Modern Recruitment Trends—A Mixed-Method Study From Germany
Source: Front Psychol. 2021 Oct 25;12:634376. doi: 10.3389/fpsyg.2021.634376 (PMC8573412; doi:10.3389/fpsyg.2021.634376)
Supplement: Supplementary Data Sheet 1 — STATA output of the calculations. [file Data_Sheet_1.PDF]

```
. ******************************************************************************
. ******************************************************************************
. **Step 1: Null models
. ******************************************************************************
. ******************************************************************************
. *1.1. Active sourcing (head) nested in companies (lpp_betnr)
. **********************************************************
. cap noisily {
. melogit head || lpp_betnr:, or nolog

Mixed-effects logistic regression               Number of obs     =      3,949
Group variable:       lpp_betnr                 Number of groups  =      1,413

                                                Obs per group:
                                                              min =          1
                                                              avg =        2.8
                                                              max =         28

Integration method: mvaghermite                 Integration pts.  =          7

                                                Wald chi2(0)      =          .
Log likelihood = -2195.4361                     Prob > chi2       =          .
------------------------------------------------------------------------------
        head | Odds Ratio   Std. Err.      z    P>|z|     [95% Conf. Interval]
-------------+----------------------------------------------------------------
       _cons |   .3107674   .0138821   -26.16   0.000      .284716    .3392025
-------------+----------------------------------------------------------------
lpp_betnr    |
   var(_cons)|   .2284684   .0830894                      .1120111    .4660057
------------------------------------------------------------------------------
LR test vs. logistic model: chibar2(01) = 12.15       Prob >= chibar2 = 0.0002
. estat ic

Akaike's information criterion and Bayesian information criterion

-----------------------------------------------------------------------------
       Model |        Obs  ll(null)  ll(model)      df         AIC        BIC
-------------+---------------------------------------------------------------
           . |      3,949         .  -2195.436       2    4394.872   4407.435
-----------------------------------------------------------------------------
               Note: N=Obs used in calculating BIC; see [R] BIC note.
. estat icc

Intraclass correlation

------------------------------------------------------------------------------
                       Level |        ICC   Std. Err.     [95% Conf. Interval]
-----------------------------+------------------------------------------------
                   lpp_betnr |   .0649365   .0220826      .0329262    .1240738
------------------------------------------------------------------------------

. *********************************************************
. *1.2. Job offers (job) nested in companies (lpp_betnr)
. *********************************************************

. cap noisily {
. melogit job || lpp_betnr:, or nolog

Mixed-effects logistic regression               Number of obs     =      2,356
Group variable:       lpp_betnr                 Number of groups  =      1,109

                                                Obs per group:
                                                              min =          1
                                                              avg =        2.1
                                                              max =         18
```

```
Integration method: mvaghermite                 Integration pts.  =          7

                                                Wald chi2(0)      =          .
Log likelihood =  -1628.847                     Prob > chi2       =          .
------------------------------------------------------------------------------
         job | Odds Ratio   Std. Err.      z    P>|z|     [95% Conf. Interval]
-------------+----------------------------------------------------------------
       _cons |   1.128411   .0487472     2.80   0.005     1.036801    1.228115
-------------+----------------------------------------------------------------
lpp_betnr    |
   var(_cons)|   .0541215      .0779                      .0032225    .9089709
------------------------------------------------------------------------------
LR test vs. logistic model: chibar2(01) = 0.56        Prob >= chibar2 = 0.2270
. estat ic

Akaike's information criterion and Bayesian information criterion

-----------------------------------------------------------------------------
       Model |        Obs  ll(null)  ll(model)      df         AIC        BIC
-------------+---------------------------------------------------------------
           . |      2,356         .  -1628.847       2    3261.694   3273.223
-----------------------------------------------------------------------------
               Note: N=Obs used in calculating BIC; see [R] BIC note.
. estat icc

Intraclass correlation

------------------------------------------------------------------------------
                       Level |        ICC   Std. Err.     [95% Conf. Interval]
-----------------------------+------------------------------------------------
                   lpp_betnr |   .0161847   .0229185      .0009786    .2164815
------------------------------------------------------------------------------


. ***************************************************************************************
. ***************************************************************************************
. **Step 2: Mixed logistic regressions
. ***************************************************************************************
. ***************************************************************************************
. *2.1. Active sourcing (head)
. ****************************
. *DV: head, IVs: age (oldhead), gender, country of birth (birthc), citizenship (citizen)
. ****************************************************************************************
. cap noisily {
. melogit head i.oldhead i.gender i.birthc i.citizen || lpp_betnr:, or nolog

Mixed-effects logistic regression               Number of obs     =      3,949
Group variable:       lpp_betnr                 Number of groups  =      1,413

                                                Obs per group:
                                                              min =          1
                                                              avg =        2.8
                                                              max =         28

Integration method: mvaghermite                 Integration pts.  =          7

                                                Wald chi2(8)      =      71.50
Log likelihood = -2157.7114                     Prob > chi2       =     0.0000
--------------------------------------------------------------------------------------------
                                    head | Odds Ratio   Std. Err.      z    P>|z|     [95% Conf. Interval]
----------------------------------------+---------------------------------------------------------------
                                         |
                                 oldhead |
                         older than 50y  |   .7825932   .0657143    -2.92   0.004      .663836    .9225956
                                         |
                                  gender |
```

female	.5547085	.0509738	-6.41	0.000	.4632819	.6641777
birthc						
born in Southern/Eastern Europe	.6196142	.1309712	-2.26	0.024	.4094476	.937658
born in Northern/Western/Central Europe	2.033797	.8507594	1.70	0.090	.8958616	4.617155
born in Asia	.8452409	.2155696	-0.66	0.510	.5127323	1.393383
born in remaining countries	1.37716	.6910599	0.64	0.524	.515052	3.68229
citizen						
German and foreign citizenship	2.334092	.5650775	3.50	0.000	1.45226	3.751385
foreign citizenship	1.024013	.2896263	0.08	0.933	.5882426	1.782603
_cons	.3943298	.022626	-16.22	0.000	.3523863	.4412658
lpp_betnr						
var(_cons)	.2115208	.0825725			.0984159	.4546124

LR test vs. logistic model: chibar2(01) = 10.20   Prob >= chibar2 = 0.0007

. estat ic

Akaike's information criterion and Bayesian information criterion

Model	Obs	ll(null)	ll(model)	df	AIC	BIC
.	3,949	.	-2157.711	10	4335.423	4398.235

Note: N=Obs used in calculating BIC; see [R] BIC note.

. estat icc

Residual intraclass correlation

Level	ICC	Std. Err.	[95% Conf.	Interval]
lpp_betnr	.0604106	.0221581	.0290459	.1214087

. margins oldhead gender birthc citizen

Predictive margins   Number of obs   =   3,949

Model VCE   : OIM

Expression   : Marginal predicted mean, predict()

	Delta-method Margin	Std. Err.	z	P>|z|	[95% Conf.	Interval]
oldhead						
younger than 50y	.2616128	.0090396	28.94	0.000	.2438956	.2793301
older than 50y	.2192725	.0115123	19.05	0.000	.1967088	.2418362
gender						
male	.2765373	.0090189	30.66	0.000	.2588605	.294214
female	.178576	.0113853	15.68	0.000	.1562613	.2008907
birthc						
born in Germany	.2505225	.0078418	31.95	0.000	.2351527	.2658922
born in Southern/Eastern Europe	.1750294	.0280745	6.23	0.000	.1200043	.2300545
born in Northern/Western/Central Europe	.3941209	.0919101	4.29	0.000	.2139805	.5742614
born in Asia	.2218507	.0411555	5.39	0.000	.1411874	.302514
born in remaining countries	.3111754	.1001389	3.11	0.002	.1149068	.507444
citizen						
German citizenship	.2426755	.0075639	32.08	0.000	.2278505	.2575004
German and foreign citizenship	.4153613	.0536078	7.75	0.000	.310292	.5204307

```
                   foreign citizenship  |   .2468301   .0486513     5.07   0.000     .1514752    .3421849
------------------------------------------------------------------------------------------------------
. ***********************
. *2.2. Job offers (job)
. ***********************
. *DV: job (job offers), IVs: age (oldjob), gender, country of birth (birthc), citizenship (citizen)
. *************************************************************************************************
. cap noisily{
. melogit job i.oldjob i.gender i.birthc i.citizen || lpp_betnr:, or nolog

Mixed-effects logistic regression               Number of obs     =      2,356
Group variable:       lpp_betnr                 Number of groups  =      1,109

                                                Obs per group:
                                                              min =          1
                                                              avg =        2.1
                                                              max =         18

Integration method: mvaghermite                 Integration pts.  =          7

                                                Wald chi2(8)      =      18.97
Log likelihood =  -1619.147                     Prob > chi2       =     0.0150
-------------------------------------------------------------------------------------------------------
                                    job | Odds Ratio   Std. Err.      z    P>|z|     [95% Conf. Interval]
----------------------------------------+--------------------------------------------------------------
                                        |
                                 oldjob |
                        older than 50y  |   1.059007     .09425     0.64   0.519     .8894945    1.260824
                                        |
                                 gender |
                                female  |   .6766381   .0659251    -4.01   0.000     .5590152    .8190101
                                        |
                                 birthc |
       born in Southern/Eastern Europe  |     .92975   .2199218    -0.31   0.758     .5848226    1.478115
born in Northern/Western/Central Europe |   .7951324   .3852114    -0.47   0.636     .3076555    2.055011
                          born in Asia  |   .7033189   .2166319    -1.14   0.253     .3845657    1.286276
           born in remaining countries  |   .4991071   .2810643    -1.23   0.217      .165521    1.504993
                                        |
                                citizen |
        German and foreign citizenship  |   1.196054   .3133173     0.68   0.494     .7157657    1.998623
                   foreign citizenship  |   1.186771   .3772058     0.54   0.590     .6365304    2.212659
                                        |
                                  _cons |   1.232682   .0756002     3.41   0.001     1.093068    1.390129
----------------------------------------+--------------------------------------------------------------
lpp_betnr                               |
                              var(_cons)|   .0668127   .0800971                       .006374    .7003356
-------------------------------------------------------------------------------------------------------
LR test vs. logistic model: chibar2(01) = 0.84        Prob >= chibar2 = 0.1804
. estat ic

Akaike's information criterion and Bayesian information criterion

-----------------------------------------------------------------------------
       Model |        Obs  ll(null)  ll(model)      df         AIC        BIC
-------------+---------------------------------------------------------------
           . |      2,356         .  -1619.147      10    3258.294   3315.941
-----------------------------------------------------------------------------
               Note: N=Obs used in calculating BIC; see [R] BIC note.
. estat icc

Residual intraclass correlation

------------------------------------------------------------------------------
                       Level |        ICC   Std. Err.     [95% Conf. Interval]
-----------------------------+------------------------------------------------
                   lpp_betnr |   .0199044    .023387      .0019337    .1755137
```

```
-------------------------------------------------------------------------------
. margins oldjob gender birthc citizen

Predictive margins                              Number of obs     =      2,356
Model VCE    : OIM

Expression   : Marginal predicted mean, predict()

------------------------------------------------------------------------------------------------------
                                        |            Delta-method
                                        |     Margin   Std. Err.      z    P>|z|     [95% Conf. Interval]
----------------------------------------+-------------------------------------------------------------
                                 oldjob |
                      younger than 50y  |   .5251564   .0129409    40.58   0.000     .4997927    .5505201
                        older than 50y  |   .5390876   .0176031    30.62   0.000     .5045862    .5735889
                                        |
                                 gender |
                                  male  |   .5545667   .0121755    45.55   0.000     .5307033    .5784302
                                female  |   .4589346   .0204125    22.48   0.000      .418927    .4989423
                                        |
                                 birthc |
                       born in Germany  |   .5340784   .0111553    47.88   0.000     .5122143    .5559424
       born in Southern/Eastern Europe  |   .5163419   .0557156     9.27   0.000     .4071413    .6255426
born in Northern/Western/Central Europe |   .4781746   .1166529     4.10   0.000     .2495391    .7068102
                         born in Asia   |   .4483826   .0735078     6.10   0.000     .3043099    .5924552
           born in remaining countries  |   .3676046   .1271329     2.89   0.004     .1184287    .6167806
                                        |
                                citizen |
                    German citizenship  |   .5274432   .0110268    47.83   0.000      .505831    .5490553
        German and foreign citizenship  |   .5706649   .0611297     9.34   0.000     .4508528    .6904769
                   foreign citizenship  |   .5688009   .0741952     7.67   0.000     .4233809    .7142209
------------------------------------------------------------------------------------------------------
. *******************************************
. **Intersectional effects
. *******************************************
. *Active sourcing (head)
. ***********************
. *DV: head, IVs: age (agehead), gender, country of birth-without remaining countries (birthco), citizenship (citizen)
. *************************************************************************************************************************
. *Estimations without intersectional effects
. *******************************************
. cap noisily{
. melogit head agehead i.gender i.birthco i.citizen || lpp_betnr:, or nolog

Mixed-effects logistic regression               Number of obs     =      3,927
Group variable:       lpp_betnr                 Number of groups  =      1,408

                                                Obs per group:
                                                              min =          1
                                                              avg =        2.8
                                                              max =         27

Integration method: mvaghermite                 Integration pts.  =          7

                                                Wald chi2(7)      =      73.03
Log likelihood =  -2142.263                     Prob > chi2       =     0.0000
-------------------------------------------------------------------------------------------------------
                                   head | Odds Ratio   Std. Err.      z    P>|z|     [95% Conf. Interval]
----------------------------------------+--------------------------------------------------------------
                                agehead |   .9852388   .0037305    -3.93   0.000     .9779543    .9925776
                                        |
                                 gender |
                                female  |   .5641501   .0519282    -6.22   0.000     .4710254    .6756862
                                        |
                                birthco |
       born in Southern/Eastern Europe  |   .6461354   .1367625    -2.06   0.039     .4267331    .9783423
```

```
born in Northern/Western/Central Europe  |   2.187026   .9234135     1.85   0.064     .9559974    5.003238
                           born in Asia  |   .8355714   .2129653    -0.70   0.481     .5070311    1.376996
                                         |
                                 citizen |
         German and foreign citizenship  |   2.181212   .5381841     3.16   0.002     1.344856    3.537693
                    foreign citizenship  |   .9635908   .2849356    -0.13   0.900     .5397476    1.720262
                                         |
                                   _cons |   .6877146   .1157994    -2.22   0.026     .4944022    .9566125
-----------------------------------------+----------------------------------------------------------------
lpp_betnr                                |
                               var(_cons)|   .2076844   .0828778                      .0950006    .4540266
----------------------------------------------------------------------------------------------------------
LR test vs. logistic model: chibar2(01) = 9.73        Prob >= chibar2 = 0.0009
. estat ic

Akaike's information criterion and Bayesian information criterion

-----------------------------------------------------------------------------
       Model |        Obs  ll(null)  ll(model)      df         AIC        BIC
-------------+---------------------------------------------------------------
           . |      3,927         .  -2142.263       9    4302.526   4359.007
-----------------------------------------------------------------------------
               Note: N=Obs used in calculating BIC; see [R] BIC note.
. estimates store headsimple2
. }

. *******************************************
. *Estimations with intersectional effects
. *******************************************
. cap noisily {
. melogit head c.agehead i.gender i.birthco i.citizen ///
>  c.agehead#gender c.agehead#birthco c.agehead#citizen ///
>  gender#birthco gender#citizen ///
>  c.agehead#gender#birthco c.agehead#gender#citizen || lpp_betnr:, or nolog

Mixed-effects logistic regression               Number of obs     =      3,927
Group variable:       lpp_betnr                 Number of groups  =      1,408

                                                Obs per group:
                                                              min =          1
                                                              avg =        2.8
                                                              max =         27

Integration method: mvaghermite                 Integration pts.  =          7

                                                Wald chi2(23)     =      82.57
Log likelihood = -2134.2337                     Prob > chi2       =     0.0000
-----------------------------------------------------------------------------------------------------------------
                                           head | Odds Ratio   Std. Err.      z    P>|z|     [95% Conf. Interval]
------------------------------------------------+----------------------------------------------------------------
                                        agehead |   .9868879   .0044668    -2.92   0.004     .9781719    .9956816
                                                |
                                         gender |
                                        female  |   .7176031   .2896965    -0.82   0.411     .3252776    1.583123
                                                |
                                        birthco |
               born in Southern/Eastern Europe  |   1.205834   1.390552     0.16   0.871     .1258037    11.55798
       born in Northern/Western/Central Europe  |   .4984111    1.35736    -0.26   0.798     .0023958    103.6877
                                  born in Asia  |   .1187963   .1557883    -1.62   0.104     .0090896    1.552602
                                                |
                                        citizen |
                German and foreign citizenship  |   3.190244   4.013017     0.92   0.356     .2710786    37.54505
                           foreign citizenship  |   .5210612   .7486167    -0.45   0.650     .0311856     8.70609
                                                |
                               gender#c.agehead |
                                        female  |   .9934648   .0090127    -0.72   0.470     .9759564    1.011287
```

```
                                               |
                              birthco#c.agehead |
               born in Southern/Eastern Europe  |   .9834121   .0266548    -0.62   0.537      .932533    1.037067
       born in Northern/Western/Central Europe  |   1.026588   .0604938     0.45   0.656     .9146128    1.152272
                                 born in Asia  |   1.048798   .0360797     1.38   0.166     .9804144    1.121951
                                               |
                              citizen#c.agehead |
                German and foreign citizenship  |   .9869941   .0309637    -0.42   0.676     .9281345    1.049586
                           foreign citizenship  |   1.018269   .0358132     0.51   0.607     .9504413    1.090938
                                               |
                                 gender#birthco |
        female#born in Southern/Eastern Europe  |   .2971093   .6865133    -0.53   0.599      .003207    27.52572
female#born in Northern/Western/Central Europe  |   7168.048   43485.06     1.46   0.143     .0491559    1.05e+09
                           female#born in Asia  |   183.0557   524.8169     1.82   0.069     .6640595    50461.45
                                               |
                                 gender#citizen |
         female#German and foreign citizenship  |   .4632295   1.138034    -0.31   0.754      .003755    57.14485
                    female#foreign citizenship  |   167.3728   555.4589     1.54   0.123     .2505003    111830.8
                                               |
                       gender#birthco#c.agehead |
        female#born in Southern/Eastern Europe  |   1.034821   .0542243     0.65   0.514     .9338185    1.146748
female#born in Northern/Western/Central Europe  |   .8482408   .1049976    -1.33   0.184     .6655108    1.081143
                           female#born in Asia  |   .8985229   .0632299    -1.52   0.128     .7827613    1.031404
                                               |
                       gender#citizen#c.agehead |
         female#German and foreign citizenship  |   1.028205   .0613627     0.47   0.641     .9147039     1.15579
                    female#foreign citizenship  |   .8679833   .0724076    -1.70   0.090     .7370615     1.02216
                                               |
                                          _cons |   .6477715   .1289946    -2.18   0.029      .438447    .9570325
-----------------------------------------------+----------------------------------------------------------------
lpp_betnr                                      |
                                      var(_cons)|   .2132386   .0843514                      .0982095    .4629968
----------------------------------------------------------------------------------------------------------------
LR test vs. logistic model: chibar2(01) = 9.97        Prob >= chibar2 = 0.0008
. estat ic

Akaike's information criterion and Bayesian information criterion

-----------------------------------------------------------------------------
       Model |        Obs  ll(null)  ll(model)      df         AIC        BIC
-------------+---------------------------------------------------------------
           . |      3,927         .  -2134.234      25    4318.467   4475.358
-----------------------------------------------------------------------------
               Note: N=Obs used in calculating BIC; see [R] BIC note.
. estimates store headsquare_inter
.

. ************************
. *Likelihood-ration test
. ************************
. lrtest headsimple2 headsquare_inter

Likelihood-ratio test                                 LR chi2(16) =     16.06
(Assumption: headsimple2 nested in headsquare_i~r)    Prob > chi2 =    0.4489
. }

. *****************************************************
. *Job offers (job)
. ******************
. *DV: job, IVs: age (agejob), gender, country of birth-without remaining countries (birthco), citizenship (citizen)
. ***************************************************************************************************************
. *Estimations without intersectional effects
. ******************************************
. cap noisily {
. melogit job agejob i.gender i.birthco i.citizen || lpp_betnr:, or nolog
```

```
Mixed-effects logistic regression               Number of obs     =      2,341
Group variable:       lpp_betnr                 Number of groups  =      1,107

                                                Obs per group:
                                                              min =          1
                                                              avg =        2.1
                                                              max =         18

Integration method: mvaghermite                 Integration pts.  =          7

                                                Wald chi2(7)      =      17.18
Log likelihood = -1609.5167                     Prob > chi2       =     0.0162
------------------------------------------------------------------------------------------------------------
                                    job | Odds Ratio   Std. Err.      z    P>|z|     [95% Conf. Interval]
----------------------------------------+-------------------------------------------------------------------
                                 agejob |   1.002036   .0040885     0.50   0.618     .9940549    1.010082
                                        |
                                 gender |
                                female  |     .68138   .0663739    -3.94   0.000     .5629542    .8247186
                                        |
                                birthco |
        born in Southern/Eastern Europe  |   .9442927   .2243389    -0.24   0.809     .5927666    1.504283
born in Northern/Western/Central Europe  |   .8280389   .4041685    -0.39   0.699     .3181093    2.155386
                          born in Asia  |   .7040314   .2167551    -1.14   0.254     .3850585    1.287234
                                        |
                                citizen |
         German and foreign citizenship  |    1.19066   .3182283     0.65   0.514      .705158    2.010429
                    foreign citizenship  |   1.115548   .3665447     0.33   0.739     .5858707    2.124097
                                        |
                                  _cons |   1.149422   .2131239     0.75   0.453     .7991925    1.653134
----------------------------------------+-------------------------------------------------------------------
lpp_betnr                               |
                              var(_cons)|   .0629878    .079156                       .0053649    .7395209
------------------------------------------------------------------------------------------------------------
LR test vs. logistic model: chibar2(01) = 0.75        Prob >= chibar2 = 0.1926
. estat ic

Akaike's information criterion and Bayesian information criterion

-----------------------------------------------------------------------------
       Model |        Obs  ll(null)  ll(model)      df         AIC        BIC
-------------+---------------------------------------------------------------
           . |      2,341         .  -1609.517       9    3237.033   3288.858
-----------------------------------------------------------------------------
               Note: N=Obs used in calculating BIC; see [R] BIC note.
. estimates store jobsimple2
. }

. *******************************************
. *Estimations with intersectional effects
. *******************************************
. cap noisily {
.  melogit job c.agejob i.gender i.birthco i.citizen ///
>  c.agejob#gender c.agejob#birthco c.agejob#citizen ///
>  gender#birthco gender#citizen ///
>  c.agejob#gender#birthco c.agejob#gender#citizen || lpp_betnr:, or nolog

Mixed-effects logistic regression               Number of obs     =      2,341
Group variable:       lpp_betnr                 Number of groups  =      1,107

                                                Obs per group:
                                                              min =          1
                                                              avg =        2.1
                                                              max =         18

Integration method: mvaghermite                 Integration pts.  =          7
```

```
                                              Wald chi2(23)     =      26.62
Log likelihood = -1603.8323                   Prob > chi2       =     0.2726
---------------------------------------------------------------------------------------------------------------------
                                          job | Odds Ratio   Std. Err.      z    P>|z|     [95% Conf. Interval]
----------------------------------------------+----------------------------------------------------------------------
                                       agejob |   .9994847   .0048739    -0.11   0.916     .9899775    1.009083
                                              |
                                       gender |
                                      female  |   .4087851   .1809217    -2.02   0.043     .1716987    .9732471
                                              |
                                      birthco |
             born in Southern/Eastern Europe  |   1.038076   1.428645     0.03   0.978     .0699469    15.40599
     born in Northern/Western/Central Europe  |   11.90579   38.92865     0.76   0.449     .0196139    7226.898
                                born in Asia  |   1.283627   1.734348     0.18   0.853     .0908562    18.13525
                                              |
                                      citizen |
              German and foreign citizenship  |   4.112816   5.853945     0.99   0.320     .2526901    66.94072
                         foreign citizenship  |   .6477873    1.06198    -0.26   0.791     .0260601    16.10234
                                              |
                              gender#c.agejob |
                                      female  |   1.011176   .0099658     1.13   0.259     .9918307    1.030898
                                              |
                             birthco#c.agejob |
             born in Southern/Eastern Europe  |   .9975148   .0316655    -0.08   0.938     .9373427     1.06155
      born in Northern/Western/Middle Europe  |   .9348891   .0692102    -0.91   0.363     .8086217    1.080873
                                born in Asia  |   .9844441   .0346935    -0.44   0.656     .9187413    1.054846
                                              |
                             citizen#c.agejob |
              German and foreign citizenship  |   .9662183   .0341553    -0.97   0.331     .9015415    1.035535
                         foreign citizenship  |    1.01214   .0395262     0.31   0.757     .9375604    1.092652
                                              |
                               gender#birthco |
      female#born in Southern/Eastern Europe  |   .0213954   .0611877    -1.34   0.179     .0000787    5.816136
 female#born in Northern/Western/Central Europe |   .0030434   .0187039    -0.94   0.346     1.79e-08    518.2382
                         female#born in Asia  |   .7931646    3.43663    -0.05   0.957     .0001627    3867.731
                                              |
                               gender#citizen |
       female#German and foreign citizenship  |   .3704964   1.105632    -0.33   0.739     .0010682    128.5088
                  female#foreign citizenship  |   164.6242    628.917     1.34   0.182     .0921791    294005.3
                                              |
                      gender#birthco#c.agejob |
      female#born in Southern/Eastern Europe  |    1.08449   .0671821     1.31   0.190     .9604953    1.224492
 female#born in Northern/Western/Central Europe |   1.153025   .1561237     1.05   0.293     .8842657    1.503469
                         female#born in Asia  |    1.00301   .1104483     0.03   0.978     .8083019     1.24462
                                              |
                      gender#citizen#c.agejob |
       female#German and foreign citizenship  |   1.041581   .0755361     0.56   0.574     .9035735    1.200668
                  female#foreign citizenship  |   .9018419   .0775138    -1.20   0.229      .762025    1.067313
                                              |
                                        _cons |   1.291898    .283818     1.17   0.244     .8398995    1.987144
----------------------------------------------+----------------------------------------------------------------------
lpp_betnr                                     |
                                    var(_cons)|    .058479   .0792148                       .0041113    .8318116
---------------------------------------------------------------------------------------------------------------------
LR test vs. logistic model: chibar2(01) = 0.64        Prob >= chibar2 = 0.2116
. estat ic

Akaike's information criterion and Bayesian information criterion

-----------------------------------------------------------------------------
       Model |        Obs  ll(null)  ll(model)      df         AIC        BIC
-------------+---------------------------------------------------------------
           . |      2,341         .  -1603.832      25    3257.665   3401.623
-----------------------------------------------------------------------------
               Note: N=Obs used in calculating BIC; see [R] BIC note.
```

```
. estimates store jobsimple_inter
.
. ************************
. *Likelihood-ration test
. ************************
. lrtest jobsimple2 jobsimple_inter

Likelihood-ratio test                                 LR chi2(16) =     11.37
(Assumption: jobsimple2 nested in jobsimple_in~r)     Prob > chi2 =    0.7862
. }
. ******************************************************************************
. *Squared age effects, depending on gender
. *****************************************
. *Active sourcing (head)
. ***********************
. cap noisily {
. melogit head c.agehead##c.agehead i.gender i.birthc i.citizen c.agehead#gender || lpp_betnr:, or nolog

Mixed-effects logistic regression               Number of obs     =      3,949
Group variable:       lpp_betnr                 Number of groups  =      1,413

                                                Obs per group:
                                                              min =          1
                                                              avg =        2.8
                                                              max =         28

Integration method: mvaghermite                 Integration pts.  =          7

                                                Wald chi2(10)     =      80.51
Log likelihood = -2151.7807                     Prob > chi2       =     0.0000
------------------------------------------------------------------------------------------------------------
                                   head | Odds Ratio   Std. Err.      z    P>|z|     [95% Conf. Interval]
----------------------------------------+-------------------------------------------------------------------
                                agehead |   1.049552   .0338622     1.50   0.134     .9852386    1.118064
                                        |
                    c.agehead#c.agehead |    .999267   .0003869    -1.89   0.058     .9985089    1.000026
                                        |
                                 gender |
                                female  |    .876211   .3410783    -0.34   0.734     .4085693    1.879108
                                        |
                                 birthc |
        born in Southern/Eastern Europe  |   .6177206    .130717    -2.28   0.023      .408007    .9352258
 born in Northern/Western/Central Europe |   2.105087   .8832857     1.77   0.076      .924931    4.791049
                           born in Asia  |   .8263443   .2109963    -0.75   0.455      .500977    1.363027
            born in remaining countries  |   1.401629   .7060591     0.67   0.503     .5222123       3.762
                                        |
                                citizen |
         German and foreign citizenship  |   2.267076   .5496178     3.38   0.001     1.409631    3.646085
                    foreign citizenship  |   1.010523   .2859445     0.04   0.970     .5803424    1.759576
                                        |
                       gender#c.agehead |
                                female  |   .9896647   .0087266    -1.18   0.239     .9727078    1.006917
                                        |
                                  _cons |    .190723   .1235176    -2.56   0.011      .053597    .6786804
----------------------------------------+-------------------------------------------------------------------
lpp_betnr                               |
                              var(_cons)|   .2118701   .0832322                       .0981023    .4575727
------------------------------------------------------------------------------------------------------------
LR test vs. logistic model: chibar2(01) = 10.09       Prob >= chibar2 = 0.0007
. estat ic

Akaike's information criterion and Bayesian information criterion

-----------------------------------------------------------------------------
       Model |        Obs  ll(null)  ll(model)      df         AIC        BIC
-------------+---------------------------------------------------------------
```

```
          . |      3,949         .  -2151.781      12    4327.561   4402.936
-----------------------------------------------------------------------------
               Note: N=Obs used in calculating BIC; see [R] BIC note.
. margins gender, at(agehead=(20(5)65))

Predictive margins                              Number of obs     =      3,949
Model VCE    : OIM

Expression   : Marginal predicted mean, predict()

1._at        : agehead         =          20

2._at        : agehead         =          25

3._at        : agehead         =          30

4._at        : agehead         =          35

5._at        : agehead         =          40

6._at        : agehead         =          45

7._at        : agehead         =          50

8._at        : agehead         =          55

9._at        : agehead         =          60

10._at       : agehead         =          65

------------------------------------------------------------------------------
             |            Delta-method
             |     Margin   Std. Err.      z    P>|z|     [95% Conf. Interval]
-------------+----------------------------------------------------------------
  _at#gender |
     1#male  |     .28206   .0335175     8.42   0.000      .216367     .347753
   1#female  |   .2210337   .0391094     5.65   0.000     .1443806    .2976868
     2#male  |   .2971355   .0217702    13.65   0.000     .2544668    .3398042
   2#female  |   .2251734   .0287576     7.83   0.000     .1688095    .2815373
     3#male  |   .3051919   .0147445    20.70   0.000     .2762932    .3340906
   3#female  |   .2232069   .0214464    10.41   0.000     .1811727     .265241
     4#male  |   .3058995   .0127732    23.95   0.000     .2808645    .3309345
   4#female  |   .2152449   .0170511    12.62   0.000     .1818254    .2486644
     5#male  |   .2992302    .012628    23.70   0.000     .2744798    .3239807
   5#female  |   .2017452   .0145385    13.88   0.000     .1732504    .2302401
     6#male  |   .2854544   .0117439    24.31   0.000     .2624368     .308472
   6#female  |   .1835132   .0130363    14.08   0.000     .1579626    .2090638
     7#male  |   .2651694   .0109954    24.12   0.000     .2436188    .2867199
   7#female  |   .1616849   .0128082    12.62   0.000     .1365813    .1867886
     8#male  |   .2393443    .013872    17.25   0.000     .2121557    .2665329
   8#female  |   .1376681   .0144679     9.52   0.000     .1093115    .1660247
     9#male  |   .2093474   .0208697    10.03   0.000     .1684436    .2502513
   9#female  |    .113022   .0174193     6.49   0.000     .0788808    .1471633
    10#male  |   .1769142   .0292862     6.04   0.000     .1195143     .234314
  10#female  |   .0892806   .0202688     4.40   0.000     .0495544    .1290067
------------------------------------------------------------------------------

. *****************
. *Job offers (job)
. *****************
. cap noisily {
. melogit job c.agejob##c.agejob i.gender i.birthc i.citizen ///
> c.agejob#gender || lpp_betnr:, or nolog

Mixed-effects logistic regression               Number of obs     =      2,356
Group variable:       lpp_betnr                 Number of groups  =      1,109
```

```
                                                Obs per group:
                                                              min =          1
                                                              avg =        2.1
                                                              max =         18

Integration method: mvaghermite                Integration pts.  =          7

                                                Wald chi2(10)     =      20.97
Log likelihood = -1618.0648                     Prob > chi2       =     0.0213
-----------------------------------------------------------------------------------------------------------
                                      job | Odds Ratio   Std. Err.      z    P>|z|     [95% Conf. Interval]
------------------------------------------+----------------------------------------------------------------
                                   agejob |   .9978791   .0346627    -0.06   0.951     .9322025    1.068183
                                          |
                        c.agejob#c.agejob |   1.000002   .0004078     0.00   0.996     .9992029    1.000802
                                          |
                                   gender |
                                  female  |   .3579893   .1514502    -2.43   0.015     .1562293     .820309
                                          |
                                   birthc |
        born in Southern/Eastern Europe  |   .9302859   .2203823    -0.31   0.760     .5847484    1.480007
 born in Northern/Western/Central Europe |   .7867155   .3815669    -0.49   0.621     .3040705    2.035453
                            born in Asia  |   .6932128   .2140049    -1.19   0.235     .3785195    1.269536
            born in remaining countries  |   .4829492   .2720493    -1.29   0.196     .1601078    1.456768
                                          |
                                  citizen |
         German and foreign citizenship  |   1.194787   .3139103     0.68   0.498     .7139229    1.999537
                    foreign citizenship  |   1.190984   .3789945     0.55   0.583      .638318    2.222159
                                          |
                          gender#c.agejob |
                                  female  |   1.014744   .0095847     1.55   0.121     .9961311    1.033704
                                          |
                                    _cons |    1.37446   .9786684     0.45   0.655     .3404428    5.549071
------------------------------------------+----------------------------------------------------------------
lpp_betnr                                 |
                              var(_cons)|   .0688821   .0804221                      .0069872    .6790651
-----------------------------------------------------------------------------------------------------------
LR test vs. logistic model: chibar2(01) = 0.88        Prob >= chibar2 = 0.1734
. estat ic

Akaike's information criterion and Bayesian information criterion

-----------------------------------------------------------------------------
       Model |        Obs  ll(null)  ll(model)      df         AIC        BIC
-------------+---------------------------------------------------------------
           . |      2,356         .  -1618.065      12     3260.13   3329.306
-----------------------------------------------------------------------------
               Note: N=Obs used in calculating BIC; see [R] BIC note.
. margins gender, at(agejob=(20(5)65))

Predictive margins                              Number of obs     =      2,356
Model VCE    : OIM

Expression   : Marginal predicted mean, predict()

1._at        : agejob          =          20

2._at        : agejob          =          25

3._at        : agejob          =          30

4._at        : agejob          =          35

5._at        : agejob          =          40
```

```
6._at        : agejob          =          45

7._at        : agejob          =          50

8._at        : agejob          =          55

9._at        : agejob          =          60

10._at       : agejob          =          65

------------------------------------------------------------------------------
             |            Delta-method
             |     Margin   Std. Err.      z    P>|z|     [95% Conf. Interval]
-------------+----------------------------------------------------------------
  _at#gender |
     1#male  |   .5658576   .0475278    11.91   0.000     .4727049    .6590104
   1#female  |   .3878065   .0605919     6.40   0.000     .2690485    .5065645
     2#male  |   .5633984   .0302404    18.63   0.000     .5041282    .6226686
   2#female  |   .4026044   .0445226     9.04   0.000     .3153417    .4898671
     3#male  |   .5609592   .0198656    28.24   0.000     .5220234    .5998951
   3#female  |   .4176016   .0334346    12.49   0.000     .3520709    .4831322
     4#male  |   .5585404   .0165969    33.65   0.000     .5260111    .5910697
   4#female  |   .4327731   .0269833    16.04   0.000     .3798868    .4856593
     5#male  |    .556142   .0165942    33.51   0.000     .5236181     .588666
   5#female  |   .4480926    .023839    18.80   0.000     .4013691    .4948162
     6#male  |   .5537644   .0160357    34.53   0.000      .522335    .5851938
   6#female  |   .4635329   .0228097    20.32   0.000     .4188267    .5082391
     7#male  |   .5514076   .0151549    36.38   0.000     .5217046    .5811105
   7#female  |   .4790655   .0246347    19.45   0.000     .4307824    .5273486
     8#male  |   .5490718   .0186935    29.37   0.000     .5124332    .5857104
   8#female  |   .4946613    .031605    15.65   0.000     .4327166     .556606
     9#male  |   .5467572   .0303628    18.01   0.000     .4872472    .6062671
   9#female  |   .5102907   .0448759    11.37   0.000     .4223355    .5982458
    10#male  |   .5444639   .0490147    11.11   0.000     .4483968     .640531
  10#female  |   .5259235   .0640793     8.21   0.000     .4003304    .6515166
------------------------------------------------------------------------------


. *************************************************************************************
. *************************************************************************************
. **Step 3: Interactions with far-right voting (rightZ)
. *************************************************************************************
. *************************************************************************************
. ** Excluding data sets with missings in far-right voting (rightZ)
. ****************************************************************
.
. keep if !missing(rightZ)
(2,0/ observations deleted)

.
. *****************************
. *3.1. Active sourcing (head)
. *****************************
. *Mixed logistic regressions in the reduced sample, but without far-right voting
. ********************************************************************************
.
. cap noisily {
. melogit head i.oldhead i.gender i.birthc i.citizen || lpp_betnr:, or nolog

Mixed-effects logistic regression               Number of obs     =      2,107
Group variable:       lpp_betnr                 Number of groups  =        594

                                                Obs per group:
                                                              min =          1
                                                              avg =        3.5
                                                              max =         28
```

```
Integration method: mvaghermite                 Integration pts.  =          7

                                                Wald chi2(8)      =      24.78
Log likelihood = -1113.6678                     Prob > chi2       =     0.0017
------------------------------------------------------------------------------------------------------------
                                       head | Odds Ratio   Std. Err.      z    P>|z|     [95% Conf. Interval]
--------------------------------------------+---------------------------------------------------------------
                                            |
                                    oldhead |
                            older than 50y  |   .8423613   .0981552    -1.47   0.141     .6703677    1.058483
                                            |
                                     gender |
                                    female  |   .6162392   .0806078    -3.70   0.000     .4768777    .7963274
                                            |
                                     birthc |
            born in Southern/Eastern Europe  |    .724275   .2052721    -1.14   0.255     .4155834     1.26226
 born in Northern/Western/Central Europe |   3.179489    1.94462     1.89   0.059     .9588499      10.543
                               born in Asia  |   .6324003   .2451393    -1.18   0.237     .2958272    1.351904
                born in remaining countries  |   2.124445   1.422653     1.13   0.260     .5717824    7.893328
                                            |
                                    citizen |
           German and foreign citizenship  |   1.421941   .5016702     1.00   0.318     .7121508    2.839168
                      foreign citizenship  |   .9431134   .3734263    -0.15   0.882     .4340404    2.049263
                                            |
                                      _cons |   .3332105   .0284173   -12.89   0.000     .2819197    .3938329
--------------------------------------------+---------------------------------------------------------------
lpp_betnr                                   |
                                 var(_cons)|   .2757278    .116464                      .1204879    .6309827
------------------------------------------------------------------------------------------------------------
LR test vs. logistic model: chibar2(01) = 9.80        Prob >= chibar2 = 0.0009
. estat ic

Akaike's information criterion and Bayesian information criterion

-----------------------------------------------------------------------------
       Model |        Obs  ll(null)  ll(model)      df         AIC        BIC
-------------+---------------------------------------------------------------
           . |      2,107         .  -1113.668      10    2247.336   2303.866
-----------------------------------------------------------------------------
               Note: N=Obs used in calculating BIC; see [R] BIC note.
. estat icc

Residual intraclass correlation

------------------------------------------------------------------------------
                      Level |        ICC   Std. Err.     [95% Conf. Interval]
----------------------------+-------------------------------------------------
                  lpp_betnr |   .0773301   .0301374        .03533      .16093
------------------------------------------------------------------------------
. }

.
. *******************************************************
. *Effect of far-right voting without interactions
. *******************************************************
. cap noisily{
. melogit head i.oldhead i.gender i.birthc i.citizen rightZ || lpp_betnr:, or nolog

Mixed-effects logistic regression               Number of obs     =      2,107
Group variable:       lpp_betnr                 Number of groups  =        594

                                                Obs per group:
                                                              min =          1
                                                              avg =        3.5
                                                              max =         28
```

```
Integration method: mvaghermite                 Integration pts.  =          7

                                                Wald chi2(9)      =      30.19
Log likelihood = -1110.6892                     Prob > chi2       =     0.0004
--------------------------------------------------------------------------------------------------------------
                                       head | Odds Ratio   Std. Err.      z    P>|z|     [95% Conf. Interval]
--------------------------------------------+-----------------------------------------------------------------
                                            |
                                    oldhead |
                            older than 50y  |   .8400176   .0980078    -1.49   0.135     .6683066    1.055847
                                            |
                                     gender |
                                    female  |   .6116701   .0801659    -3.75   0.000     .4731057    .7908176
                                            |
                                     birthc |
             born in Southern/Eastern Europe  |   .6897504   .1954747    -1.31   0.190     .3957875    1.202048
 born in Northern/Western/Central Europe |    3.17881   1.943672     1.89   0.059     .9589595    10.53729
                               born in Asia  |   .6053568   .2349207    -1.29   0.196     .2829345    1.295201
                 born in remaining countries  |   2.039403   1.364759     1.06   0.287     .5493928    7.570478
                                            |
                                    citizen |
          German and foreign citizenship  |   1.372322   .4840106     0.90   0.370     .6874514    2.739494
                     foreign citizenship  |   .9317293   .3677906    -0.18   0.858       .42982    2.019728
                                            |
                                     rightZ |    .861389   .0536129    -2.40   0.017     .7624659    .9731464
                                      _cons |   .3371054   .0286481   -12.80   0.000     .2853831    .3982017
--------------------------------------------+-----------------------------------------------------------------
lpp_betnr                                   |
                                  var(_cons)|   .2741953   .1156254                      .1199822    .6266183
--------------------------------------------------------------------------------------------------------------
LR test vs. logistic model: chibar2(01) = 9.92        Prob >= chibar2 = 0.0008
. }

. ********************************************************************************
. *Interactions with far-right voting, successively
. **************************************************
. *Interaction with age (oldhead)
. *******************************
.
. cap noisily{
. melogit head i.oldhead i.gender i.birthc i.citizen rightZ oldhead#c.rightZ || lpp_betnr:, or nolog

Mixed-effects logistic regression               Number of obs     =      2,107
Group variable:       lpp_betnr                 Number of groups  =        594

                                                Obs per group:
                                                              min =          1
                                                              avg =        3.5
                                                              max =         28

Integration method: mvaghermite                 Integration pts.  =          7

                                                Wald chi2(10)     =      33.86
Log likelihood = -1108.7149                     Prob > chi2       =     0.0002
--------------------------------------------------------------------------------------------------------------
                                       head | Odds Ratio   Std. Err.      z    P>|z|     [95% Conf. Interval]
--------------------------------------------+-----------------------------------------------------------------
                                            |
                                    oldhead |
                            older than 50y  |   .8449231    .098844    -1.44   0.150      .671798    1.062663
                                            |
                                     gender |
                                    female  |   .6076059   .0798538    -3.79   0.000      .469628    .7861221
                                            |
                                     birthc |
```

```
       born in Southern/Eastern Europe  |   .6941707   .1970536    -1.29   0.198     .3979573    1.210866
 born in Northern/Western/Central Europe |    3.48719   2.145134     2.03   0.042     1.044388    11.64366
                   born in Asia  |   .5957646   .2314839    -1.33   0.183     .2781896    1.275876
           born in remaining countries  |   2.051575   1.377952     1.07   0.285      .550013    7.652472
                                        |
                               citizen |
        German and foreign citizenship  |   1.328166   .4699133     0.80   0.422     .6638835    2.657129
                   foreign citizenship  |   .8985233   .3561296    -0.27   0.787     .4131956    1.953903
                                        |
                                rightZ |   .7875733   .0618134    -3.04   0.002     .6752797    .9185405
                                        |
                      oldhead#c.rightZ |
                       older than 50y  |    1.26532   .1496135     1.99   0.047     1.003582     1.59532
                                        |
                                 _cons |    .335446   .0287199   -12.76   0.000     .2836255    .3967345
----------------------------------------+----------------------------------------------------------------
lpp_betnr                               |
                            var(_cons)|   .2845235   .1178377                       .1263531    .6406932
---------------------------------------------------------------------------------------------------------
LR test vs. logistic model: chibar2(01) = 10.39       Prob >= chibar2 = 0.0006
. }

.
. *************************
. *Interaction with gender
. *************************

. cap noisily{
. melogit head i.oldhead i.gender i.birthc i.citizen rightZ ///
> gender#c.rightZ || lpp_betnr:, or nolog

Mixed-effects logistic regression               Number of obs     =      2,107
Group variable:       lpp_betnr                 Number of groups  =        594

                                                Obs per group:
                                                              min =          1
                                                              avg =        3.5
                                                              max =         28

Integration method: mvaghermite                 Integration pts.  =          7

                                                Wald chi2(10)     =      30.74
Log likelihood = -1110.5141                     Prob > chi2       =     0.0006
---------------------------------------------------------------------------------------------------------
                                  head | Odds Ratio   Std. Err.      z    P>|z|     [95% Conf. Interval]
----------------------------------------+----------------------------------------------------------------
                                        |
                               oldhead |
                       older than 50y  |   .8391516    .097952    -1.50   0.133     .6675471     1.05487
                                        |
                                gender |
                               female  |   .6148593   .0806624    -3.71   0.000     .4754535    .7951397
                                        |
                                birthc |
       born in Southern/Eastern Europe  |   .6919017   .1961159    -1.30   0.194     .3969866    1.205905
 born in Northern/Western/Central Europe |   3.215773   1.968684     1.91   0.056     .9686858    10.67549
                   born in Asia  |    .606915   .2357512    -1.29   0.199      .283456    1.299482
           born in remaining countries  |   2.046241   1.371175     1.07   0.285      .550264     7.60926
                                        |
                               citizen |
        German and foreign citizenship  |   1.372359    .484274     0.90   0.370     .6872243    2.740547
                   foreign citizenship  |   .9274214   .3663964    -0.19   0.849     .4275559    2.011691
                                        |
                                rightZ |    .843629   .0606004    -2.37   0.018     .7328366    .9711712
                                        |
                       gender#c.rightZ |
```

```
                              female  |    1.07984   .1395685     0.59   0.552       .83819    1.391159
                                      |
                               _cons  |   .3367966    .028658   -12.79   0.000     .2850619    .3979206
-----------------------------------------+----------------------------------------------------------------
lpp_betnr                                |
                           var(_cons)|   .2768165   .1160455                      .1217183    .6295468
----------------------------------------------------------------------------------------------------------
LR test vs. logistic model: chibar2(01) = 10.07       Prob >= chibar2 = 0.0008
. }


. *******************************************
. *Interaction with country of birth (birthc)
. *******************************************

. cap noisily{
. melogit head i.oldhead i.gender i.birthc i.citizen rightZ ///
> birthc#c.rightZ || lpp_betnr:, or nolog

Mixed-effects logistic regression               Number of obs     =      2,107
Group variable:       lpp_betnr                 Number of groups  =        594

                                                Obs per group:
                                                              min =          1
                                                              avg =        3.5
                                                              max =         28

Integration method: mvaghermite                 Integration pts.  =          7

                                                Wald chi2(13)     =      31.62
Log likelihood = -1109.4385                     Prob > chi2       =     0.0027
----------------------------------------------------------------------------------------------------------
                                    head | Odds Ratio   Std. Err.      z    P>|z|     [95% Conf. Interval]
-----------------------------------------+----------------------------------------------------------------
                                         |
                                 oldhead |
                         older than 50y  |   .8374362   .0978848    -1.52   0.129      .665975    1.053042
                                         |
                                  gender |
                                 female  |   .6079116   .0797898    -3.79   0.000     .4700221    .7862536
                                         |
                                  birthc |
        born in Southern/Eastern Europe  |   .6036446   .2178676    -1.40   0.162     .2975537    1.224609
 born in Northern/Western/Central Europe |   2.950093   1.896824     1.68   0.092     .8366384     10.4024
                          born in Asia   |   .4121854   .2357805    -1.55   0.121     .1343333    1.264741
            born in remaining countries  |   2.564114   2.060161     1.17   0.241     .5309231    12.38349
                                         |
                                 citizen |
         German and foreign citizenship  |   1.350252   .4789725     0.85   0.397      .673705    2.706201
                    foreign citizenship  |   .9591904   .3826045    -0.10   0.917     .4389097    2.096208
                                         |
                                  rightZ |   .8728635   .0552405    -2.15   0.032     .7710398    .9881342
                                         |
                         birthc#c.rightZ |
        born in Southern/Eastern Europe  |   .7630577    .323966    -0.64   0.524     .3320235    1.753662
 born in Northern/Western/Central Europe |   .7080694   .4742681    -0.52   0.606     .1905185    2.631567
                          born in Asia   |   .4167274   .3292482    -1.11   0.268     .0885797    1.960513
            born in remaining countries  |   1.872678   2.194836     0.54   0.592     .1882906    18.62505
                                         |
                                   _cons |   .3377822   .0287559   -12.75   0.000     .2858727    .3991176
-----------------------------------------+----------------------------------------------------------------
lpp_betnr                                |
                           var(_cons)|   .2771782   .1165451                      .1215779    .6319223
----------------------------------------------------------------------------------------------------------
LR test vs. logistic model: chibar2(01) = 9.99        Prob >= chibar2 = 0.0008
. }
```

```
. ***************************************
. *Interaction with citizenship (citizen)
. ***************************************
.
. cap noisily{
. melogit head i.oldhead i.gender i.birthc i.citizen rightZ ///
> citizen#c.rightZ || lpp_betnr:, or nolog

Mixed-effects logistic regression               Number of obs     =      2,107
Group variable:       lpp_betnr                 Number of groups  =        594

                                                Obs per group:
                                                              min =          1
                                                              avg =        3.5
                                                              max =         28

Integration method: mvaghermite                 Integration pts.  =          7

                                                Wald chi2(11)     =      30.33
Log likelihood = -1110.5984                     Prob > chi2       =     0.0014
------------------------------------------------------------------------------------------------------------
                                   head | Odds Ratio   Std. Err.      z    P>|z|     [95% Conf. Interval]
----------------------------------------+-------------------------------------------------------------------
                                        |
                                oldhead |
                        older than 50y  |   .8391524   .0979237    -1.50   0.133      .667592    1.054801
                                        |
                                 gender |
                                female  |   .6120498   .0802281    -3.75   0.000     .4733805      .79134
                                        |
                                 birthc |
        born in Southern/Eastern Europe  |   .6864236    .194888    -1.33   0.185     .3934783    1.197467
 born in Northern/Western/Central Europe |   3.382254   2.152583     1.91   0.056     .9715659    11.77444
                          born in Asia  |   .6066194   .2353159    -1.29   0.198     .2836113    1.297505
            born in remaining countries  |   2.040261   1.366651     1.06   0.287     .5489285    7.583253
                                        |
                                citizen |
         German and foreign citizenship  |   1.441309   .5746381     0.92   0.359      .659766    3.148648
                   foreign citizenship  |   .8749977   .3833098    -0.30   0.761     .3707845    2.064868
                                        |
                                 rightZ |   .8620707    .054367    -2.35   0.019     .7618357    .9754937
                                        |
                       citizen#c.rightZ |
         German and foreign citizenship  |   1.118318   .4854382     0.26   0.797     .4776136    2.618508
                   foreign citizenship  |   .8563291   .3957907    -0.34   0.737     .3461146    2.118661
                                        |
                                  _cons |   .3373589   .0286519   -12.79   0.000     .2856272    .3984599
----------------------------------------+-------------------------------------------------------------------
lpp_betnr                               |
                             var(_cons)|   .2722704   .1153884                       .1186481    .6247989
------------------------------------------------------------------------------------------------------------
LR test vs. logistic model: chibar2(01) = 9.81        Prob >= chibar2 = 0.0009
. }


. ****************************************************
. *Interactions with far-right voting, simultaneously
. ****************************************************
.
. cap noisily{
. melogit head i.oldhead i.gender i.birthc i.citizen rightZ ///
> oldhead#c.rightZ gender#c.rightZ birthc#c.rightZ citizen#c.rightZ || lpp_betnr:, or nolog

Mixed-effects logistic regression               Number of obs     =      2,107
```

```
Group variable:       lpp_betnr                Number of groups  =        594

                                               Obs per group:
                                                             min =          1
                                                             avg =        3.5
                                                             max =         28

Integration method: mvaghermite                Integration pts.  =          7

                                               Wald chi2(17)     =      35.81
Log likelihood = -1107.2045                    Prob > chi2       =     0.0049
------------------------------------------------------------------------------------------------------------
                                        head | Odds Ratio   Std. Err.      z    P>|z|     [95% Conf. Interval]
----------------------------------------+-------------------------------------------------------------------
                                             |
                                     oldhead |
                              older than 50y  |   .8399613   .0985096    -1.49   0.137     .6674696    1.057029
                                             |
                                      gender |
                                     female  |   .6067853   .0799673    -3.79   0.000     .4686585    .7856219
                                             |
                                      birthc |
            born in Southern/Eastern Europe  |   .5588466   .2170055    -1.50   0.134     .2610736    1.196251
   born in Northern/Western/Central Europe |   2.930973   2.185437     1.44   0.149     .6797134    12.63857
                                born in Asia  |   .4038613   .2342411    -1.56   0.118     .1295783    1.258729
                    born in remaining countries  |   2.402135   1.994754     1.06   0.291     .4717938    12.23045
                                             |
                                     citizen |
             German and foreign citizenship  |   1.612954   .7118817     1.08   0.279     .6791127    3.830912
                       foreign citizenship  |   1.017871   .5207313     0.03   0.972      .373446    2.774328
                                             |
                                      rightZ |   .7853794   .0677765    -2.80   0.005     .6631666    .9301143
                                             |
                            oldhead#c.rightZ |
                              older than 50y  |   1.253718   .1494137     1.90   0.058     .9925595    1.583591
                                             |
                             gender#c.rightZ |
                                     female  |   1.057103   .1372372     0.43   0.669     .8196171    1.363401
                                             |
                             birthc#c.rightZ |
            born in Southern/Eastern Europe  |   .6424732   .3254812    -0.87   0.382     .2380294    1.734121
   born in Northern/Western/Central Europe |   .5931287   .5377264    -0.58   0.565     .1003357    3.506246
                                born in Asia  |   .4093112   .3313464    -1.10   0.270     .0837505    2.000414
                    born in remaining countries  |   1.560003   1.968246     0.35   0.724     .1315738    18.49615
                                             |
                            citizen#c.rightZ |
             German and foreign citizenship  |   1.558645   .8789923     0.79   0.431     .5160781     4.70738
                       foreign citizenship  |   1.249358   .8382655     0.33   0.740     .3354034    4.653787
                                             |
                                       _cons |   .3363259   .0288245   -12.71   0.000     .2843211     .397843
----------------------------------------+-------------------------------------------------------------------
lpp_betnr                                    |
                                  var(_cons)|   .2854376   .1184132                       .1265878    .6436213
------------------------------------------------------------------------------------------------------------
LR test vs. logistic model: chibar2(01) = 10.37       Prob >= chibar2 = 0.0006
. }

. *********************************************************************************************************
. *3.2. Job offers (job)
. **********************
. *Mixed logistic regressions in the reduced sample, but without far-right voting
. *********************************************************************************
. . cap noisily {
. xtmelogit job i.oldjob i.gender i.birthc i.citizen || lpp_betnr:, or mle

Refining starting values:
```

```
Iteration 0:   log likelihood = -904.70763
Iteration 1:   log likelihood = -893.67661
Iteration 2:   log likelihood = -892.71984

Performing gradient-based optimization:

Iteration 0:   log likelihood = -892.71984
Iteration 1:   log likelihood = -892.71892
Iteration 2:   log likelihood = -892.71892

Mixed-effects logistic regression               Number of obs     =      1,296
Group variable: lpp_betnr                       Number of groups  =        508

                                                Obs per group:
                                                              min =          1
                                                              avg =        2.6
                                                              max =         18

Integration points =   7                        Wald chi2(8)      =       7.90
Log likelihood = -892.71892                     Prob > chi2       =     0.4435

-----------------------------------------------------------------------------------------------------------
                                    job | Odds Ratio   Std. Err.      z    P>|z|     [95% Conf. Interval]
----------------------------------------+------------------------------------------------------------------
                                 oldjob |
                         older than 50y  |   .9605128   .1134544    -0.34   0.733     .7620096    1.210726
                                        |
                                 gender |
                                female  |   .7197127   .0964327    -2.45   0.014     .5534883    .9358577
                                        |
                                 birthc |
       born in Southern/Eastern Europe  |   .7283021     .22502    -1.03   0.305     .3974842    1.334453
born in Northern/Western/Central Europe |   .7454831   .4767385    -0.46   0.646      .212859     2.61086
                         born in Asia  |   .7644941   .3333385    -0.62   0.538     .3252588    1.796881
           born in remaining countries  |   .7197122   .5508113    -0.43   0.667     .1605891    3.225535
                                        |
                                citizen |
        German and foreign citizenship  |    1.37688   .5100477     0.86   0.388     .6661608    2.845856
                   foreign citizenship  |   1.357501   .5840774     0.71   0.477     .5841207    3.154841
                                        |
                                  _cons |   1.214615   .1028298     2.30   0.022     1.028906    1.433843
-----------------------------------------------------------------------------------------------------------

------------------------------------------------------------------------------
  Random-effects Parameters  |   Estimate   Std. Err.     [95% Conf. Interval]
-----------------------------+------------------------------------------------
lpp_betnr: Identity          |
                   sd(_cons) |   .3083916   .1704671      .1043736    .9112009
------------------------------------------------------------------------------
LR test vs. logistic model: chibar2(01) = 1.07        Prob >= chibar2 = 0.1505
. }

. ******************************************************
. *Effect of far-right voting without interactions
. ******************************************************
. cap noisily {
. melogit job i.oldjob i.gender i.birthc i.citizen rightZ || lpp_betnr:, or nolog

Mixed-effects logistic regression               Number of obs     =      1,296
Group variable:       lpp_betnr                 Number of groups  =        508

                                                Obs per group:
                                                              min =          1
                                                              avg =        2.6
                                                              max =         18
```

```
Integration method: mvaghermite                 Integration pts.  =          7

                                                Wald chi2(9)      =       8.84
Log likelihood = -892.20331                     Prob > chi2       =     0.4521
-------------------------------------------------------------------------------------------------------
                                     job | Odds Ratio   Std. Err.      z    P>|z|     [95% Conf. Interval]
-----------------------------------------+-------------------------------------------------------------
                                         |
                                  oldjob |
                        older than 50y  |   .9599228   .1136536    -0.35   0.730     .7611235    1.210647
                                         |
                                  gender |
                                 female  |   .7190723   .0966343    -2.45   0.014      .552563    .9357577
                                         |
                                  birthc |
       born in Southern/Eastern Europe  |   .7138999   .2214453    -1.09   0.277      .388689     1.31121
 born in Northern/Western/Central Europe |   .7494863   .4800692    -0.45   0.653     .2135712    2.630175
                          born in Asia  |   .7447828   .3260063    -0.67   0.501     .3158215    1.756376
            born in remaining countries  |   .7055891   .5408674    -0.45   0.649       .15706    3.169845
                                         |
                                 citizen |
        German and foreign citizenship  |   1.349163   .5014764     0.81   0.420     .6511447    2.795447
                   foreign citizenship  |   1.346818   .5810269     0.69   0.490     .5782214    3.137064
                                         |
                                  rightZ |   .9403507   .0573035    -1.01   0.313     .8344858    1.059646
                                   _cons |   1.220331   .1040342     2.34   0.020     1.032552    1.442259
-----------------------------------------+-------------------------------------------------------------
lpp_betnr                                |
                              var(_cons)|   .1044714   .1068515                      .0140736    .7755164
-------------------------------------------------------------------------------------------------------
LR test vs. logistic model: chibar2(01) = 1.28        Prob >= chibar2 = 0.1294
. }
.
. ********************************************************************************
. *Interactions with far-right voting, successively
. **************************************************
. *Interaction with age (oldjob)
. *******************************
. cap noisily {
. melogit job i.oldjob i.gender i.birthc i.citizen rightZ ///
> oldjob#c.rightZ || lpp_betnr:, or nolog

Mixed-effects logistic regression               Number of obs     =      1,296
Group variable:       lpp_betnr                 Number of groups  =        508

                                                Obs per group:
                                                              min =          1
                                                              avg =        2.6
                                                              max =         18

Integration method: mvaghermite                 Integration pts.  =          7

                                                Wald chi2(10)     =       9.49
Log likelihood = -891.86157                     Prob > chi2       =     0.4866
-------------------------------------------------------------------------------------------------------
                                     job | Odds Ratio   Std. Err.      z    P>|z|     [95% Conf. Interval]
-----------------------------------------+-------------------------------------------------------------
                                         |
                                  oldjob |
                        older than 50y  |   .9587739   .1135692    -0.36   0.722     .7601324    1.209325
                                         |
                                  gender |
                                 female  |   .7196023   .0967666    -2.45   0.014     .5528783     .936603
                                         |
                                  birthc |
```

```
       born in Southern/Eastern Europe  |   .7139242   .2215923    -1.09   0.278     .3885535    1.311757
 born in Northern/Western/Central Europe |   .7717228   .4953951    -0.40   0.686     .2193038    2.715667
                           born in Asia  |   .7431037   .3253832    -0.68   0.498     .3150165    1.752934
            born in remaining countries  |   .7130709   .5476651    -0.44   0.660     .1582625    3.212827
                                        |
                                citizen |
         German and foreign citizenship  |   1.336831   .4974303     0.78   0.435      .644685     2.77208
                    foreign citizenship  |   1.325109   .5726932     0.65   0.515     .5680341    3.091212
                                        |
                                 rightZ |   .9038411   .0702671    -1.30   0.193     .7760993    1.052609
                                        |
                        oldjob#c.rightZ |
                        older than 50y  |   1.103239   .1312038     0.83   0.409     .8738555    1.392835
                                        |
                                  _cons |   1.220981   .1042028     2.34   0.019     1.032914    1.443289
----------------------------------------+----------------------------------------------------------------
lpp_betnr                               |
                             var(_cons)|    .105941   .1072091                        .0145772    .7699351
---------------------------------------------------------------------------------------------------------
LR test vs. logistic model: chibar2(01) = 1.31        Prob >= chibar2 = 0.1265
. }

. ************************
. *Interaction with gender
. ************************
.
. cap noisily {
. melogit job i.oldjob i.gender i.birthc i.citizen rightZ ///
> gender#c.rightZ || lpp_betnr:, or nolog

Mixed-effects logistic regression               Number of obs     =      1,296
Group variable:       lpp_betnr                 Number of groups  =        508

                                                Obs per group:
                                                              min =          1
                                                              avg =        2.6
                                                              max =         18

Integration method: mvaghermite                 Integration pts.  =          7

                                                Wald chi2(10)     =       9.26
Log likelihood = -891.96668                     Prob > chi2       =     0.5074
---------------------------------------------------------------------------------------------------------
                                    job | Odds Ratio   Std. Err.      z    P>|z|     [95% Conf. Interval]
----------------------------------------+----------------------------------------------------------------
                                        |
                                 oldjob |
                        older than 50y  |   .9591168   .1135877    -0.35   0.724     .7604386    1.209703
                                        |
                                 gender |
                                female  |   .7190909   .0967225    -2.45   0.014     .5524481    .9360005
                                        |
                                 birthc |
       born in Southern/Eastern Europe  |   .7100999     .22026    -1.10   0.270     .3866271    1.304207
 born in Northern/Western/Central Europe |    .743437   .4760692    -0.46   0.643     .2119174    2.608085
                           born in Asia  |    .745866   .3262341    -0.67   0.503     .3164856    1.757792
             born in remaning countries  |   .7002172   .5360889    -0.47   0.642     .1561528      3.1399
                                        |
                                citizen |
         German and foreign citizenship  |   1.347873   .5008262     0.80   0.422     .6506832    2.792081
                    foreign citizenship  |    1.35034   .5821806     0.70   0.486     .5800414    3.143598
                                        |
                                 rightZ |   .9647368   .0688503    -0.50   0.615     .8388055    1.109574
                                        |
                        gender#c.rightZ |
                                female  |   .9152604   .1180638    -0.69   0.492     .7107947    1.178542
```

```
                                         |
                                   _cons |   1.220716   .1040436     2.34   0.019     1.032917     1.44266
-----------------------------------------+----------------------------------------------------------------
lpp_betnr                                |
                              var(_cons)|   .1044821    .107007                      .0140369    .7777024
----------------------------------------------------------------------------------------------------------
LR test vs. logistic model: chibar2(01) = 1.27        Prob >= chibar2 = 0.1297
. }

. *******************************************
. *Interaction with country of birth (birthc)
. *******************************************
.
. cap noisily {
. melogit job i.oldjob i.gender i.birthc i.citizen rightZ ///
> birthc#c.rightZ || lpp_betnr:, or nolog

Mixed-effects logistic regression               Number of obs     =      1,296
Group variable:       lpp_betnr                 Number of groups  =        508

                                                Obs per group:
                                                              min =          1
                                                              avg =        2.6
                                                              max =         18

Integration method: mvaghermite                 Integration pts.  =          7

                                                Wald chi2(13)     =      13.03
Log likelihood = -888.98613                     Prob > chi2       =     0.4455
----------------------------------------------------------------------------------------------------------
                                     job | Odds Ratio   Std. Err.      z    P>|z|     [95% Conf. Interval]
-----------------------------------------+----------------------------------------------------------------
                                         |
                                  oldjob |
                         older than 50y  |   .9702967   .1153854    -0.25   0.800      .768567    1.224976
                                         |
                                  gender |
                                 female  |   .7223881   .0974556    -2.41   0.016     .5545453    .9410315
                                         |
                                  birthc |
         born in Southern/Eastern Europe  |   .6510659   .2403282    -1.16   0.245     .3158062    1.342237
 born in Northern/Western/Central Europe  |   .9163702   .6665259    -0.12   0.904     .2202651     3.81238
                           born in Asia  |   .5968644   .3349183    -0.92   0.358     .1987198     1.79271
             born in remaining countries  |   3.190174   4.698216     0.79   0.431     .1779251    57.19939
                                         |
                                 citizen |
        German and foreign citizenship  |   1.390671   .5258448     0.87   0.383     .6627737    2.917987
                   foreign citizenship  |   1.264907   .5628425     0.53   0.597      .528812    3.025629
                                         |
                                  rightZ |    .936636   .0583624    -1.05   0.293     .8289569    1.058302
                                         |
                         birthc#c.rightZ |
         born in Southern/Eastern Europe  |   .7740556   .3487684    -0.57   0.570     .3200692    1.871977
  born in Northern/Western/Middle Europe  |   3.275731   3.645717     1.07   0.286      .369799    29.01689
                           born in Asia  |   .6014009    .446571    -0.68   0.493     .1403147    2.577655
                  born in other countries  |   16.07375   28.95455     1.54   0.123       .47076     548.826
                                         |
                                   _cons |   1.214473   .1039469     2.27   0.023     1.026913    1.436291
-----------------------------------------+----------------------------------------------------------------
lpp_betnr                                |
                              var(_cons)|   .1116257   .1084334                      .0166305    .7492435
----------------------------------------------------------------------------------------------------------
LR test vs. logistic model: chibar2(01) = 1.44        Prob >= chibar2 = 0.1153
. }

. ***************************************
```

```
. *Interaction with citizenship (citizen)
. **************************************
.
. cap noisily {
. melogit job i.oldjob i.gender i.birthc i.citizen rightZ ///
> citizen#c.rightZ || lpp_betnr:, or nolog

Mixed-effects logistic regression               Number of obs     =      1,296
Group variable:       lpp_betnr                 Number of groups  =        508

                                                Obs per group:
                                                              min =          1
                                                              avg =        2.6
                                                              max =         18

Integration method: mvaghermite                 Integration pts.  =          7

                                                Wald chi2(11)     =      13.27
Log likelihood =  -889.2643                     Prob > chi2       =     0.2761
-------------------------------------------------------------------------------------------------------------
                                     job | Odds Ratio   Std. Err.      z    P>|z|     [95% Conf. Interval]
-----------------------------------------+-------------------------------------------------------------------
                                         |
                                  oldjob |
                         older than 50y  |   .9542695   .1135237    -0.39   0.694     .7558032    1.204851
                                         |
                                  gender |
                                 female  |    .719998   .0972827    -2.43   0.015     .5524857    .9382997
                                         |
                                  birthc |
        born in Southern/Eastern Europe  |   .6886324   .2155378    -1.19   0.233     .3728778    1.271769
 born in Northern/Western/Central Europe |   .4613583     .32297    -1.11   0.269     .1169935    1.819344
                           born in Asia  |   .7329208    .321812    -0.71   0.479     .3099632    1.733022
            born in remaining countries  |   .7280381    .573288    -0.40   0.687     .1555543    3.407425
                                         |
                                 citizen |
         German and foreign citizenship  |   1.351788   .7153116     0.57   0.569      .479167    3.813555
                    foreign citizenship  |   2.507764   1.361375     1.69   0.090     .8653662    7.267306
                                         |
                                  rightZ |   .9242045    .057287    -1.27   0.204     .8184765     1.04359
                                         |
                        citizen#c.rightZ |
         German and foreign citizenship  |   .9516589    .706403    -0.07   0.947     .2221501     4.07677
                    foreign citizenship  |   4.403208   3.089531     2.11   0.035     1.113063    17.41882
                                         |
                                   _cons |    1.22683   .1053017     2.38   0.017     1.036868    1.451593
-----------------------------------------+-------------------------------------------------------------------
lpp_betnr                                |
                              var(_cons)|   .1150981   .1109034                       .0174136     .760761
-------------------------------------------------------------------------------------------------------------
LR test vs. logistic model: chibar2(01) = 1.46        Prob >= chibar2 = 0.1134
. }

. ****************************************************
. *Interactions with far-right voting, simultaneously
. ****************************************************
.
. cap noisily {
. melogit job i.oldjob i.gender i.birthc i.citizen rightZ ///
> oldjob#c.rightZ gender#c.rightZ birthc#c.rightZ citizen#c.rightZ || lpp_betnr:, or nolog

Mixed-effects logistic regression               Number of obs     =      1,296
Group variable:       lpp_betnr                 Number of groups  =        508

                                                Obs per group:
                                                              min =          1
```

```
                                                avg =        2.6
                                                max =         18

Integration method: mvaghermite                 Integration pts.  =          7

                                                Wald chi2(17)     =      17.43
Log likelihood = -885.93855                     Prob > chi2       =     0.4255
------------------------------------------------------------------------------------------------------------
                                        job | Odds Ratio   Std. Err.      z    P>|z|     [95% Conf. Interval]
-----------------------------------------+------------------------------------------------------------------
                                         |
                                  oldjob |
                         older than 50y  |   .9552207   .1141671    -0.38   0.701     .7557339    1.207365
                                         |
                                  gender |
                                 female  |   .7194882   .0975859    -2.43   0.015     .5515352    .9385862
                                         |
                                  birthc |
        born in Southern/Eastern Europe  |   .4561479   .2162606    -1.66   0.098     .1801148    1.155213
 born in Northern/Western/Middle Europe  |   .3348615   .3051122    -1.20   0.230     .0561416     1.99731
                          born in Asia   |   .5930646   .3338784    -0.93   0.353     .1967432    1.787739
                born in other countries  |   2.714512   4.177348     0.65   0.516     .1329788    55.41165
                                         |
                                 citizen |
         German and foreign citizenship  |   1.355907   .7737376     0.53   0.594     .4430966    4.149171
                    foreign citizenship  |   3.431624   2.372867     1.78   0.075     .8849504    13.30701
                                         |
                                  rightZ |   .9141847   .0807348    -1.02   0.310     .7688851    1.086942
                                         |
                         oldjob#c.rightZ |
                         older than 50y  |   1.125168   .1356279     0.98   0.328     .8884103    1.425022
                                         |
                         gender#c.rightZ |
                                 female  |   .9126638   .1188472    -0.70   0.483     .7070779    1.178025
                                         |
                         birthc#c.rightZ |
        born in Southern/Eastern Europe  |   .4342157   .2819417    -1.28   0.199     .1216213    1.550248
 born in Northern/Western/Central Europe |   .5431972   .8122475    -0.41   0.683     .0289833    10.18044
                          born in Asia   |   .6108679   .4566939    -0.66   0.510      .141116    2.644346
            born in remaining countries  |   10.17204   19.31761     1.22   0.222     .2459805    420.6446
                                         |
                        citizen#c.rightZ |
         German and foreign citizenship  |   .9593945   .7935558    -0.05   0.960     .1896411    4.853578
                    foreign citizenship  |   7.431738   7.319898     2.04   0.042     1.078198    51.22502
                                         |
                                   _cons |   1.226635   .1056335     2.37   0.018     1.036127    1.452172
-----------------------------------------+------------------------------------------------------------------
lpp_betnr                                |
                               var(_cons)|   .1199696    .112149                      .0192025    .7495216
------------------------------------------------------------------------------------------------------------
LR test vs. logistic model: chibar2(01) = 1.57        Prob >= chibar2 = 0.1051
. }


. **********************************************************************************************************
. **********************************************************************************************************
. **Step 4: Robustness check
. **********************************************************************************************************
. **********************************************************************************************************

. clear
. use "$data/LPPsimple.dta", clear

. ****************************************
. *excluding missing in controls
. ****************************************
```

```
.
. keep if !missing(vocational)
(163 observations deleted)

. keep if !missing(school)
(37 observations deleted)

. keep if !missing(child)
(1,608 observations deleted)

. keep if !missing(earnZ)
(285 observations deleted)

.
. *************************************
. *4.1. Active sourcing (head)
. ****************************
. *Mixed logistic regressions in the reduced sample, but without control variables
. *********************************************************************************
.
. cap noisily {
.  melogit head i.oldhead i.gender i.birthc i.citizen || lpp_betnr:, or nolog

Mixed-effects logistic regression               Number of obs     =      2,088
Group variable:       lpp_betnr                 Number of groups  =        992

                                                Obs per group:
                                                              min =          1
                                                              avg =        2.1
                                                              max =         19

Integration method: mvaghermite                 Integration pts.  =          7

                                                Wald chi2(8)      =      47.29
Log likelihood =  -1141.647                     Prob > chi2       =     0.0000
--------------------------------------------------------------------------------------------------------
                                   head | Odds Ratio   Std. Err.      z    P>|z|     [95% Conf. Interval]
----------------------------------------+---------------------------------------------------------------
                                        |
                                oldhead |
                        older than 50y  |   .8328296   .1031353    -1.48   0.140     .6533497    1.061614
                                        |
                                 gender |
                                female  |   .4638466    .065125    -5.47   0.000     .3522605      .61078
                                        |
                                 birthc |
        born in Southern/Eastern Europe  |   .6027313   .1755844    -1.74   0.082     .3405307     1.06682
 born in Northern/Western/Central Europ  |   2.627477   1.594302     1.59   0.111     .7999195    8.630411
                           born in Asia  |   .6796617   .2419596    -1.08   0.278     .3382712    1.365591
            born in remaining countries  |   2.004181    1.40279     0.99   0.321     .5083398    7.901688
                                        |
                                citizen |
         German and foreign citizenship  |   2.960308     1.0246     3.14   0.002     1.502191    5.833759
                   foreign citizenship  |   .7967832   .3239684    -0.56   0.576     .3591249    1.767807
                                        |
                                  _cons |   .3886523   .0298653   -12.30   0.000     .3343121    .4518251
----------------------------------------+---------------------------------------------------------------
lpp_betnr                               |
                             var(_cons)|   .2957408   .1482915                       .1106875    .7901764
--------------------------------------------------------------------------------------------------------
LR test vs. logistic model: chibar2(01) = 6.14        Prob >= chibar2 = 0.0066
. }

. ***************************************************
. *mixed logistic regressions with control variables
. ***************************************************
```

```
.
. cap noisily {
.  melogit head i.oldhead i.gender i.birthc i.citizen ///
>  i.school i.vocational earnZ child || lpp_betnr:, or nolog

Mixed-effects logistic regression               Number of obs     =      2,088
Group variable:       lpp_betnr                 Number of groups  =        992

                                                Obs per group:
                                                              min =          1
                                                              avg =        2.1
                                                              max =         19

Integration method: mvaghermite                 Integration pts.  =          7

                                                Wald chi2(17)     =     155.84
Log likelihood = -1066.5723                     Prob > chi2       =     0.0000
------------------------------------------------------------------------------------------------------------
                                   head | Odds Ratio   Std. Err.      z    P>|z|     [95% Conf. Interval]
----------------------------------------+-------------------------------------------------------------------
                                        |
                                oldhead |
                       older than 50y  |   .6374544   .0905517    -3.17   0.002      .482541    .8421006
                                        |
                                 gender |
                               female  |   .6619121   .1067026    -2.56   0.010     .4825959    .9078562
                                        |
                                 birthc |
        born in Southern/Eastern Europe  |   .6200413   .1868282    -1.59   0.113     .3435106    1.119183
 born in Northern/Western/Central Europe |   1.801525   1.140182     0.93   0.352     .5210943    6.228222
                          born in Asia  |   .8146634   .3001669    -0.56   0.578     .3956842    1.677289
            born in remaining countries  |   1.430742    1.04876     0.49   0.625     .3401066     6.01877
                                        |
                                citizen |
         German and foreign citizenship  |   3.287539   1.163704     3.36   0.001     1.642735    6.579218
                    foreign citizenship  |    .860514   .3600943    -0.36   0.720     .3789314    1.954138
                                        |
                                 school |
                Intermediate secondary  |   .9916638   .1694229    -0.05   0.961     .7094782    1.386085
                   Technical secondary  |   1.115486    .246636     0.49   0.621     .7232055    1.720546
                       Upper secondary  |   1.004672    .225513     0.02   0.983     .6470831    1.559871
                                        |
                             vocational |
                     vocational school  |   1.921471   .3920698     3.20   0.001     1.288099    2.866281
                     technical college  |   1.992265   .3098983     4.43   0.000     1.468731    2.702416
            advanced technical college  |   1.947476   .4259003     3.05   0.002     1.268585    2.989677
                            university  |   2.838414   .6806906     4.35   0.000     1.773969    4.541565
                                        |
                                  earnZ |   1.513139   .1063751     5.89   0.000     1.318374    1.736677
                                  child |   .9038132   .0612747    -1.49   0.136     .7913541    1.032254
                                  _cons |   .2394683   .0412979    -8.29   0.000     .1707862    .3357711
----------------------------------------+-------------------------------------------------------------------
lpp_betnr                               |
                             var(_cons)|   .2726647   .1569118                       .088264    .8423145
------------------------------------------------------------------------------------------------------------
LR test vs. logistic model: chibar2(01) = 4.60        Prob >= chibar2 = 0.0160

.  margins, dydx(gender oldhead birthc citizen)

Average marginal effects                        Number of obs     =      2,088
Model VCE    : OIM

Expression   : Marginal predicted mean, predict()
dy/dx w.r.t. : 1.oldhead 1.gender 1.birthc 2.birthc 3.birthc 4.birthc 2.citizen 3.citizen

-----------------------------------------------------------------------------------------------------------
```

```
                                        |            Delta-method
                                        |      dy/dx   Std. Err.      z    P>|z|     [95% Conf. Interval]
----------------------------------------+----------------------------------------------------------------
                                oldhead |
                        older than 50y  |  -.0694799   .0206997    -3.36   0.001    -.1100507   -.0289092
                                        |
                                 gender |
                                female  |  -.0640671   .0238646    -2.68   0.007    -.1108409   -.0172934
                                        |
                                 birthc |
       born in Southern/Eastern Europe  |  -.0704558    .040196    -1.75   0.080    -.1492385    .0083268
born in Northern/Western/Central Europe |   .1059652   .1230636     0.86   0.389    -.1352351    .3471655
                         born in Asia   |  -.0320609   .0553191    -0.58   0.562    -.1404842    .0763625
           born in remaining countries  |   .0622486    .134726     0.46   0.644    -.2018095    .3263066
                                        |
                                citizen |
        German and foreign citizenship  |   .2271521   .0735923     3.09   0.002     .0829139    .3713903
                  foreign citizenship   |  -.0233227   .0630363    -0.37   0.711    -.1468715    .1002262
---------------------------------------------------------------------------------------------------------
Note: dy/dx for factor levels is the discrete change from the base level.
. }

. **********************
. *4.2. Job offers (job)
. **********************
. *Mixed logistic regressions in the reduced sample, but without control variables
. *******************************************************************************
.
. cap noisily {
. xtmelogit job i.oldjob i.gender i.birthc i.citizen || lpp_betnr:, or mle

Refining starting values:

Iteration 0:   log likelihood =  -865.3127
Iteration 1:   log likelihood = -855.75506
Iteration 2:   log likelihood = -854.27597

Performing gradient-based optimization:

Iteration 0:   log likelihood = -854.27597
Iteration 1:   log likelihood = -854.16655
Iteration 2:   log likelihood = -854.16511
Iteration 3:   log likelihood = -854.16511

Mixed-effects logistic regression               Number of obs     =      1,250
Group variable: lpp_betnr                       Number of groups  =        745

                                                Obs per group:
                                                              min =          1
                                                              avg =        1.7
                                                              max =         11

Integration points =   7                        Wald chi2(8)      =      13.95
Log likelihood = -854.16511                     Prob > chi2       =     0.0832

---------------------------------------------------------------------------------------------------------
                                    job | Odds Ratio   Std. Err.      z    P>|z|     [95% Conf. Interval]
----------------------------------------+----------------------------------------------------------------
                                 oldjob |
                        older than 50y  |   1.044988   .1323041     0.35   0.728     .8153463    1.339308
                                        |
                                 gender |
                                female  |   .7537499    .106481    -2.00   0.045     .5714516    .9942031
                                        |
                                 birthc |
        born in Southern/Eastern Europe |   1.100564   .3529576     0.30   0.765     .5869888    2.063483
```

```
born in Northern/Western/Central Europe |   .3044759   .2162611    -1.67   0.094     .0756775    1.225008
                           born in Asia |   .4376305   .1759581    -2.06   0.040     .1990055    .9623879
            born in remaining countries |   .2034442   .1767127    -1.83   0.067     .0370755    1.116358
                                        |
                                citizen |
        German and foreign citizenship  |   1.208608   .4460979     0.51   0.608     .5862816     2.49152
                   foreign citizenship  |   1.410477   .6263448     0.77   0.439     .5907128    3.367872
                                        |
                                  _cons |   1.273688   .0991257     3.11   0.002     1.093498    1.483572
--------------------------------------------------------------------------------------------------------

------------------------------------------------------------------------------
  Random-effects Parameters  |   Estimate   Std. Err.     [95% Conf. Interval]
-----------------------------+------------------------------------------------
lpp_betnr: Identity          |
                  sd(_cons) |   3.09e-08   .5159253             0           .
------------------------------------------------------------------------------
LR test vs. logistic model: chibar2(01) = 0.00        Prob >= chibar2 = 1.0000
. }


. *****************************************************
. *Mixed logistic regressions with control variables
. *****************************************************
.
. cap noisily {
. xtmelogit job i.oldjob i.gender i.birthc i.citizen ///
>  i.school i.vocational earnZ child || lpp_betnr:, or mle

Refining starting values:

Iteration 0:   log likelihood = -862.75957
Iteration 1:   log likelihood = -853.11172
Iteration 2:   log likelihood = -851.63701

Performing gradient-based optimization:

Iteration 0:   log likelihood = -851.63701
Iteration 1:   log likelihood = -851.52845
Iteration 2:   log likelihood = -851.52581
Iteration 3:   log likelihood = -851.52581

Mixed-effects logistic regression               Number of obs     =      1,250
Group variable: lpp_betnr                       Number of groups  =        745

                                                Obs per group:
                                                              min =          1
                                                              avg =        1.7
                                                              max =         11

Integration points =   7                        Wald chi2(17)     =      18.98
Log likelihood = -851.52581                     Prob > chi2       =     0.3297

--------------------------------------------------------------------------------------------------------
                                    job | Odds Ratio   Std. Err.      z    P>|z|     [95% Conf. Interval]
----------------------------------------+---------------------------------------------------------------
                                 oldjob |
                         older than 50y |   1.071751   .1509277     0.49   0.623     .8132514    1.412418
                                        |
                                 gender |
                                female  |   .7673165   .1233186    -1.65   0.099     .5599825    1.051416
                                        |
                                 birthc |
       born in Southern/Eastern Europe  |   1.141399   .3683109     0.41   0.682      .606413    2.148357
born in Northern/Western/Central Europe |   .3019241   .2160606    -1.67   0.094     .0742622    1.227519
                          born in Asia  |    .456953   .1860808    -1.92   0.054     .2057048    1.015076
```

```
           born in remaining countries  |   .2026388    .177611    -1.82   0.069     .0363624    1.129256
                                        |
                               citizen  |
       German and foreign citizenship  |   1.202029   .4468551     0.49   0.621     .5800694    2.490862
                   foreign citizenship  |   1.395344   .6212519     0.75   0.454     .5830411    3.339362
                                        |
                                school  |
              Intermediate secondary  |   .9521409   .1678292    -0.28   0.781     .6740065     1.34505
                 Technical secondary  |    1.13567    .262671     0.55   0.582     .7217322    1.787014
                     Upper secondary  |   1.058428   .2409057     0.25   0.803     .6775198    1.653487
                                        |
                            vocational  |
                   vocational school  |   .8864647   .1888993    -0.57   0.572     .5838167    1.346004
                   technical college  |   1.036271   .1636205     0.23   0.821      .760456    1.412123
          advanced technical college  |   1.193903    .267459     0.79   0.429     .7696304    1.852063
                          university  |    .813465   .1929838    -0.87   0.384     .5109784    1.295016
                                        |
                                 earnZ  |   .9920406   .0617136    -0.13   0.898     .8781674     1.12068
                                 child  |   1.024083   .0714004     0.34   0.733     .8932819    1.174038
                                 _cons  |   1.231929    .218613     1.18   0.240     .8700318    1.744361
------------------------------------------------------------------------------------------------------------

------------------------------------------------------------------------------
  Random-effects Parameters  |   Estimate   Std. Err.     [95% Conf. Interval]
-----------------------------+------------------------------------------------
lpp_betnr: Identity          |
                  sd(_cons) |   1.01e-07   .5304034             0           .
------------------------------------------------------------------------------
LR test vs. logistic model: chibar2(01) = 0.00        Prob >= chibar2 = 1.0000

. margins, dydx(gender oldjob birthc citizen)

Average marginal effects                        Number of obs     =      1,250

Expression   : Linear prediction, fixed portion, predict(xb)
dy/dx w.r.t. : 1.oldjob 1.gender 1.birthc 2.birthc 3.birthc 4.birthc 2.citizen 3.citizen

------------------------------------------------------------------------------------------------------------
                                        |            Delta-method
                                        |      dy/dx   Std. Err.      z    P>|z|     [95% Conf. Interval]
----------------------------------------+-------------------------------------------------------------------
                                oldjob  |
                        older than 50y  |   .0692939   .1408235     0.49   0.623     -.206715    .3453028
                                        |
                                gender  |
                                female  |  -.2648559   .1607141    -1.65   0.099    -.5798497    .0501379
                                        |
                                birthc  |
       born in Southern/Eastern Europe  |   .1322546   .3226838     0.41   0.682    -.5001941    .7647032
born in Northern/Western/Central Europe |   -1.19758   .7156123    -1.67   0.094    -2.600154    .2049947
                         born in Asia  |  -.7831748   .4072209    -1.92   0.054    -1.581313    .0149635
           born in remaining countries  |   -1.59633   .8764903    -1.82   0.069     -3.31422    .1215594
                                        |
                               citizen  |
       German and foreign citizenship  |   .1840107   .3717508     0.49   0.621    -.5446075    .9126289
                   foreign citizenship  |   .3331411    .445232     0.75   0.454    -.5394975     1.20578
------------------------------------------------------------------------------------------------------------
Note: dy/dx for factor levels is the discrete change from the base level.
. }
. *********************************************************************************************************
. *********************************************************************************************************
```
